# Phospholipid Saturation Modulates Cholesterol Partitioning and Heat Transport in Lipid Bilayers under Thermal Gradients

**DOI:** 10.1021/acs.langmuir.5c06531

**Published:** 2026-05-21

**Authors:** Zhibo Deng, Mona W. Qiu, Fionn Carman, John M. Seddon, Fernando Bresme

**Affiliations:** Department of Chemistry, 4615 Imperial College London, Molecular Sciences Research Hub, 80 Wood Lane, London, W12 0BZ, U.K.

## Abstract

Lipid compositional asymmetry across the two leaflets of the bilayer is a defining feature of biological membranes, critically influencing their chemical and physical properties. Understanding the factors that modulate this asymmetry is essential for elucidating the mechanisms that regulate membrane and membrane protein function. Experimental evidence has demonstrated heat generation within cells, and localized heating strategies are increasingly used in thermal therapies for cancer. Additionally, electroporation techniques, which use electric pulses to make membranes permeable, can also create thermal gradients. These findings highlight the importance of understanding membrane behavior under nonequilibrium conditions. Here, we employ nonequilibrium molecular dynamics simulations with the coarse-grained MARTINI 3 force field to investigate cholesterol partitioning in lipid bilayers under thermal gradients. We explore bilayers with varying degrees of lipid saturation and cholesterol content. Our results reveal that cholesterol exhibits thermophobic behavior, preferentially accumulating in colder regions of the bilayer, a trend that aligns with previous atomistic simulations. This thermophobicity is most pronounced in bilayers composed of saturated lipids and at low cholesterol mole fractions. We further show that bilayer thermal conductivity decreases with increasing cholesterol content, while saturated phospholipid bilayers exhibit higher thermal conductance than their unsaturated counterparts. Our findings indicate that lipid composition and cholesterol levels together modulate both mass and thermal transport in lipid membranes exposed to thermal stress.

## Graphical abstract

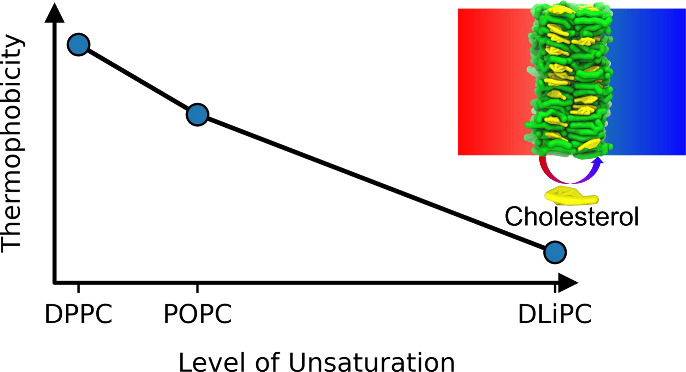

## Introduction

Lipid bilayer compositional asymmetry is a fundamental feature of nearly all biological membranes,^1,2^ and governs critical cellular functions, from immune responses to molecular transport.^3,4^ Bilayer asymmetry can arise through several dynamic processes, such as the lateral diffusion of lipids with distinct compositions,^3^ translocation of lipid rafts,^5^ and *flip-flop* processes, including ATP-driven translocation and transverse lipid diffusion.^6,7^ Lipid flip-flop plays a vital role in controlling dynamic asymmetry, in which the spontaneous translocation of lipids between membrane leaflets depends strongly on lipid species and bilayer phase behavior.

Cholesterol accounts for approximately 30% of the lipid composition in mammalian cells, making it a key component of the cell membrane. Furthermore, cholesterol modulates membrane stability, fluidity, and structure,^8–11^ and exhibits rapid flip-flop (microsecond to submillisecond time scales)^12,13^ compared to bulkier and more strongly polar lipids such as 1,2-dipalmitoyl-*sn*-glycero-3-phosphocholine (DPPC), which require hours or days for transverse movement.^14–16^

The degree of cholesterol asymmetry in mammalian cells remains an open question. A brief review of the potential role of cholesterol in modulating membrane asymmetry in relation to biomembrane properties has recently appeared.^17^ Recent studies indicate that high cholesterol content can stabilize membrane lipid asymmetry.^18^ A thermodynamic analysis of asymmetric ternary lipid bilayers^19^ has shown that cholesterol plays two crucial roles. On the one hand, it controls lateral demixing of bilayers containing mixtures of saturated and unsaturated phospholipids. On the other hand, by rapidly flipping across the bilayer in response to differential stress between the two monolayer leaflets of the membrane, it directly couples lateral stress to mixing thermodynamics. Atomistic molecular dynamics simulations by Heberle and Doktorova^20^ have found that the effect of cholesterol on lipid packing density within differentially stressed asymmetric bilayers is different from that on symmetric bilayers. Those authors proposed a number of approaches, based on simulating experimental data from simulation trajectories, which could be used in future to access these effects experimentally. It should also be noted that cholesterol exhibits a tendency for negative monolayer spontaneous curvature, suggesting that it can favor or stabilize negatively curved membrane environments. Curvature–composition coupling may bias sterol distribution in heterogeneous membranes,^21^ despite the higher concentration of saturated lipids in the outer leaflet of the mammalian plasma membrane.^22^ Cholesterol asymmetry is thus expected to vary with local membrane properties, influencing lipid packing. One factor of particular interest in our work is how cholesterol distribution responds under nonequilibrium conditions, such as those arising from stationary or transient thermal gradients.

Heat generation from metabolic processes has been observed in living cells,^23–25^ with temperature differences of about 1 °C between the nucleus and the cytoplasm. Given the typical size of a mammalian cell (tens of micrometers), a 1 °C temperature difference can create significant thermal gradients within biological environments. An alternative perspective based on basic heat-diffusion scaling proposes that single-cell thermogenesis would yield small steady-state temperature rises, offering a contrasting view on the existence of multi-Kelvin intracellular temperature heterogeneities.^26^ Early studies of nerve conduction^27^ indicate that heat generation and absorption are linked to membrane processes,^28,29^ suggesting that heat transport is a common feature of biological systems.

Local thermal gradients can also be generated using nanomaterials, including plasmonic and magnetic materials.^30,31^ Intense submicrometer-scale heating can induce hyperthermia and irreversible cellular damage.^32^ This phenomenon is exploited in thermal therapy strategies for cancer treatment.^33–35^ Membrane electroporation via electric pulses is also used in drug delivery.^36,37^ It has been suggested that these electric pulses can cause significant thermal gradients in membranes, reaching values on the order of ∼ 10^7^ K/m.^38^

The thermal transport of biological macromolecules and assemblies, such as proteins and lipid membranes, has been investigated using computational simulations. Studies employing nonequilibrium molecular dynamics and vibrational energy transfer methods predict protein thermal conductivities ranging from 0.1–0.3 W/(K m),^39–42^ while simulations of lipid bilayers report values between 0.2 and 0.5 W/(K m).^43,44^ Recent measurements^45^ suggest that the thermal conductivity of lipid bilayers is comparable to values typically observed in biological tissues. These studies indicate that biological media and tissues are generally slightly less efficient heat conductors than water, which has a thermal conductivity of approximately 0.6 W/(K m) at room temperature.^46^

Previous studies suggested that endogenous metabolic processes can result in heat generation, which is an inherent feature of biological systems.^23–25,27–29^ External perturbations can create localized thermal gradients which can potentially influence membrane properties.^31–38^ Recent advances in nanotechnology and electroporation techniques enable precise thermal manipulation at cellular and subcellular scales, opening new therapeutic avenues. Consequently, investigating thermal effects in biological systems is essential for elucidating the mechanisms that govern heat transport and cellular processes.

In an earlier study, we demonstrated, using nonequilibrium molecular dynamics simulations, that thermal gradients perpendicular to the plane of 50:50 DPPC:CHOL lipid bilayers induce an enrichment of cholesterol in the cold region, indicating that cholesterol is thermophobic.^47^ These investigations were conducted with an atomistic force field. Here, we extend our nonequilibrium studies to investigate the dependence of cholesterol thermophobicity on lipid bilayer composition, using the coarse-grained MARTINI 3 force field.^48^ We focus on thermodynamic states corresponding to the liquid-disordered *L*_*d*_ phase (low cholesterol content) and the liquid-ordered *L*_*o*_ phase (high cholesterol content).

A central aspect of this work is the use of a coarse-grained force field to probe temperature-gradient-driven cholesterol partitioning. While coarse-graining integrates out intramolecular degrees of freedom and may therefore affect absolute transport coefficients, MARTINI 3 has been extensively parametrized to reproduce key structural and thermodynamic features of lipid membranes, including lipid packing, phase behavior, and cholesterol–lipid interactions across broad composition ranges.^48^ In the present context, the thermodiffusive redistribution of cholesterol reflects an effective free-energy bias between the hot and cold leaflets that is governed primarily by these interactions. To test our analysis, we benchmark against our prior atomistic NEMD results for 50:50 DPPC:CHOL bilayers,^47^ showing that MARTINI 3 reproduces both the direction of cholesterol thermophobicity and a comparable Soret coefficient for this reference system. This supports the use of the coarse-grained model to examine systematic compositional trends across lipid saturation and cholesterol content, as reported here.

Our results show that cholesterol thermophobicity increases with the degree of phospholipid saturation and is more pronounced in the *L*_*d*_ phase, when cholesterol content is below 40 mol %. We quantify thermophobicity using the Soret coefficient and provide microscopic insights into the origins of the observed behavior.

## Methods

In this study, we investigated coarse-grained models of six different phosphatidylcholine (PC) lipids with varying degrees of saturation: 1) DLiPC (di-C16:2 or di-C18:2 dilinoleoyl PC); 2) POPC (C16:0/C18:1 1-palmitoyl-2-oleoyl PC); 3) DPPC (di-C16:0 dipalmitoyl or di-C18:0 distearoyl PC); 4) DLPC (di-C12:0 dilauroyl or di-C14:0 dimyristoyl PC); 5) DBPC (di-C20:0 diarachidoyl or di-C22:0 dibehenoyl PC); 6) DXPC (di-C24:0 dilignoceroyl or di-C26:0 dihexacosanoyl PC). The coarse-grained representations for these lipids are shown in Figure 1. All pre-equilibrated lipid bilayers were generated using CHARMM-GUI,^49–52^ and the coarse-graining approach and the interactions follow exactly the MARTINI 3 force field approach.^48^ Martini 3 maps atoms to beads with multiple sizes (regular, small, tiny), whose self- and cross-interactions are explicitly reparameterized and balanced across resolutions to ensure consistent structural and thermodynamic properties. For the coarse-grained DPPC, POPC and DLiPC used here, the hydrocarbon tails are represented with the same number of tail beads; C1 (light teal) and C4h (yellow) beads represent the saturated and unsaturated segments, respectively. Each initial symmetric bilayer consisted of 500 lipids (250 per leaflet) and was solvated with nonpolarizable coarse-grained water beads, equivalent to approximately 100–160 water molecules per lipid. We examined cholesterol (CHOL):phospholipid (PC) composition with overall bilayer mole fractions between 0 to 50 mol %, hence covering both the liquid-disordered *L*_*d*_ and liquid-ordered *L*_*o*_ phases. For the DLPC, DBPC and DXPC bilayers, we considered a single cholesterol composition (50 mol %) to isolate the effect of saturated acyl-chain at fixed sterol loading. This high-cholesterol fraction also helps maintain a well-defined fluid (liquid-ordered) regime across the imposed thermal gradients, thereby avoiding additional phase-state variability that would otherwise complicate the comparison.

**Figure 1:**
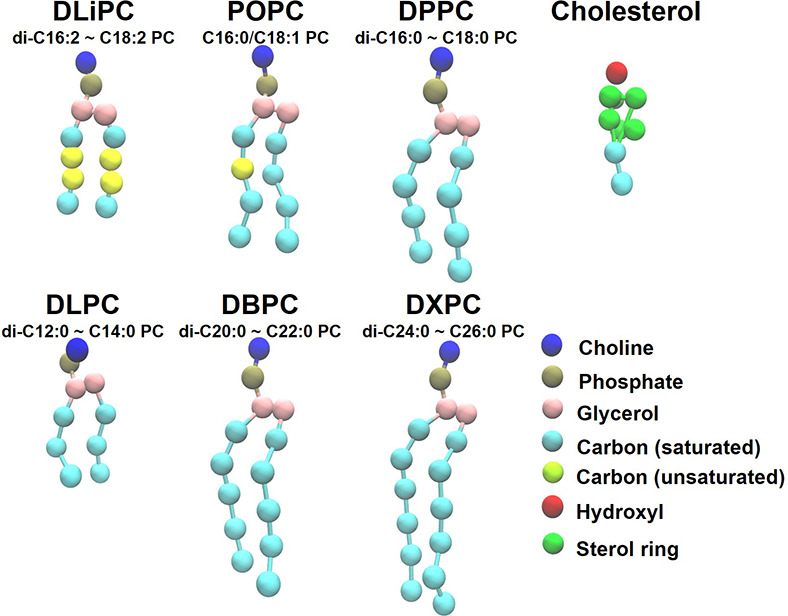
MARTINI 3 coarse-grained models of the six PC lipids and cholesterol investigated in this work.

All simulations were performed using GROMACS version 2024.1.^53,54^ Each bilayer underwent energy minimization using the steepest descent algorithm, employing soft-core potentials described by the Beutler function,^55^ until convergence was reached at a maximum force of *F*_max_ < 10 kJ /(mol nm), where *F*_max_ denotes the largest residual force on any atom in the system. The equations of motion were integrated using the leapfrog algorithm, and periodic boundary conditions were applied in all three Cartesian directions. Further simulation details are provided in the Supporting Information (SI).

The equilibrated single-bilayer systems were replicated along the *z*-axis to create two independent bilayers within the simulation box. The dimensions of the final nonequilibrium molecular dynamics (NEMD) simulation boxes and their components are listed in Table S1. To apply a heat flux, we used the boundary-driven nonequilibrium molecular dynamics approach.^56,57^ The two-bilayer configuration provides a symmetric, fully periodic NEMD geometry with thermostat slabs located in bulk water regions. Only thermostat water beads are restrained in the *z* direction. The lipids are unrestrained and the bilayer center of mass did not show systematic drift during the NEMD as shown in Figure S17.

The thermostat regions were defined in the initial configuration by selecting water beads located at the edges and center of the simulation box, each with a thickness of 0.2 nm. The motion of these water beads was restrained in the *z*-direction using a harmonic potential with a force constant of 10^3^ kJ/(mol nm^2^), while no restraints were applied in the *x*- or *y*-directions (parallel to the bilayer plane). These restrained water beads served as thermostats: the cold thermostat was maintained at 300 K, while temperatures ranging from 325–425 K were applied to the hot thermostats using the Nosé-Hoover thermostat.^58,59^ Both the cold and the hot regions contained the same number of water beads. Figure 2 in the results section shows an example of the simulation setup used in this study. Although the hot thermostat temperature can reach 425 K, it is applied only in the boundary water slabs. The temperature drop across the bilayer is much smaller (Table S3), and was chosen to obtain statistically converged steady states within accessible simulation times. The single bilayers were pre-equilibrated for ∼100 ns, followed by the equilibration step of 200 ns at 1 bar pressure and a temperature equal to the average of the hot and cold thermostat temperatures, after bilayer duplication and a further NEMD production time of 1 *μs* and 2 *μs* was performed for systems at different levels of saturation and chain lengths, respectively. As discussed in the results section and in the SI, the stationary state is reached in times below 100 ns.

**Figure 2:**
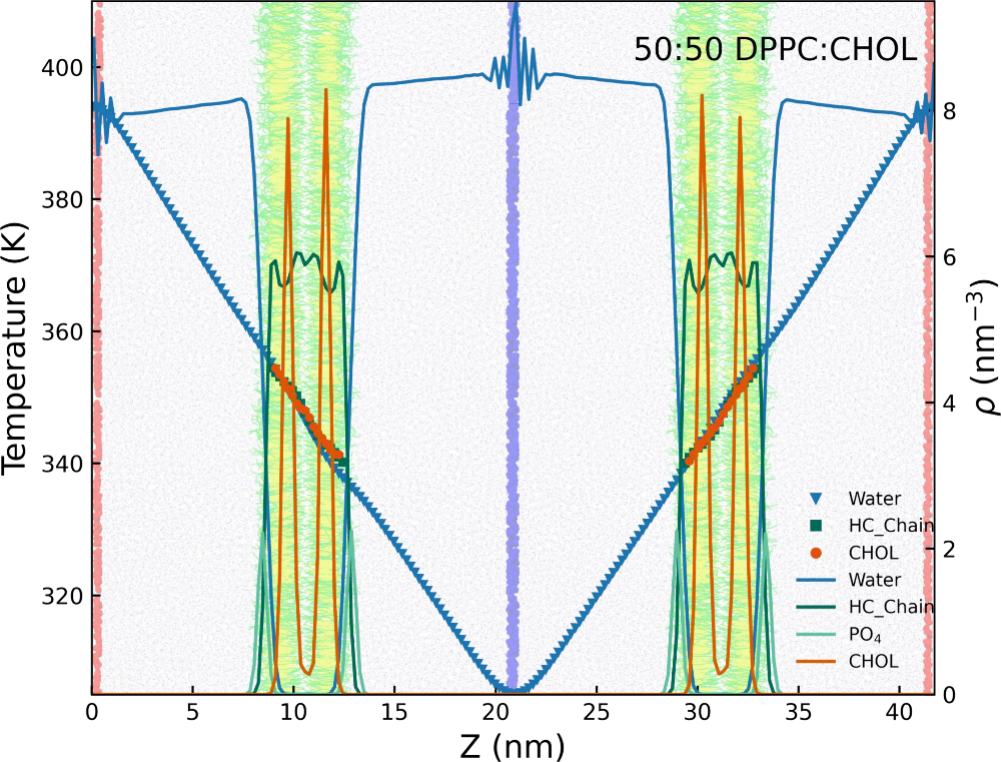
Temperature (markers and left vertical axis) and bead density (in beads per nm^–3^) profiles (lines and right vertical axis) for selected groups in a DPPC bilayer with 50 mol % cholesterol mole fraction verlaid with system snapshot. “HC_Chain” in the legend denotes the hydrocarbon chains of the PC lipid, CHOL the cholesterol molecules and Water the water beads. The density oscillations observed near the thermostat regions are caused by the water layers restrained by harmonic potentials (blue: cold thermostat, red: hot thermostat in the background), which act as semipermeable walls breaking the fluid’s translational symmetry. These oscillations are confined to areas far from the bilayer and therefore do not affect the cholesterol flip-flop motion.

We also performed constant-pressure temperature-annealing simulations to determine the gel-to-liquid phase transition temperatures of DPPC and POPC bilayers. The pressure was maintained at 1 bar. The annealing process was performed at a rate of 0.4 K/ns, spanning temperature ranges of 280 to 340 K for DPPC:CHOL and 265 to 335 K for POPC:CHOL. For each system, ten independent replicas were generated to obtain statistical averages. Additionally, a separate set of equilibrium simulations was performed for the DPPC:CHOL systems to compute two-dimensional radial distribution functions (RDFs) over temperatures from 280 to 340 K, each simulation running for 50 ns.

The thermal transport properties were determined from the steady-state heat flux, *J*_*q*_. The thermal conductivity, λ, was calculated using Fourier’s law,

$$J_{q}=-\lambda \nabla T$$ 1

where ∇*T* is the thermal gradient in the membrane region, obtained from the simulated steady state heat flux and temperature gradient, the latter by linear regression of the bilayer temperature profile. The interfacial thermal conductance, *G*, was calculated using

G=λ/δ 2

where δ represents the bilayer thickness, computed directly from the simulations as the mean separation between the phospholipid head groups (see “phosphate” bead in Figure 1). A detailed description of the thermal transport calculations is provided in the Supporting Information, which also includes additional details on the analysis of the simulation trajectories.

## Results and Discussion

We conducted an initial equilibrium simulation analysis of DPPC and POPC lipid bilayers to investigate the melting behavior of the bilayers. This investigation was performed to ensure that the temperature ranges used in the NEMD simulations targeted thermodynamic states in either the liquid disordered *L*_*d*_ or the liquid ordered *L*_*o*_ phases. For the temperature-annealing (heating/cooling) runs, we initialized the system below the melting temperature in the solid-ordered (*S*_*o*_, gel) state, to identify the *S*_*o*_ → *L*_*d*_ transition and its hysteresis. The transition from the *S*_*o*_ to the *L*_*d*_ phase was assessed through temperature-dependent calculations of the area per PC molecule (*a*_PC_) and the chain order parameter (*S*_chain_). For pure DPPC bilayers, *a*_DPPC_ increased from 0.478 ± 0.001 nm^2^ at 280 K to 0.634 ± 0.016 nm^2^ at 340 K (see Figure S26 in the Supporting Information), which is consistent with literature values.^44,60,61^ We observed a significant hysteresis at low cholesterol overall mole fractions (<40 mol %), confirming that the transition is first-order. This hysteresis decreased progressively with increasing cholesterol content and disappeared completely at ≥ 40 mol %. Above this threshold, *a*_DPPC_ showed negligible temperature dependence, in agreement with previous MARTINI 2 simulations.^61^ The melting temperature (*T*_*m*_) of MARTINI 3 DPPC, estimated from the analysis of the hysteresis cycles (Figure S6) and eq 4 in the Supporting Information, was found to be between 306–308 K, which is in good agreement with the experimental value of 314 K.^62,63^ We performed similar simulations for POPC bilayers (see Figure S11) but we did not observe hysteresis loops in the temperature interval 265–335 K. This suggests that the solid–liquid phase transition occurs at lower temperatures, consistent with the lower temperature found in experiments, at ∼271 K.^64^ For DLiPC, which contains two cis double bonds per acyl chain, we did not attempt to determine *T*_*m*_ explicitly. The high degree of unsaturation leads to increased fluidity and reduced packing efficiency, implying an even lower *T*_*m*_ outside the temperature range relevant to our NEMD simulations.

We report additional results in the SI, including the chain order parameter (*S*_chain_) (see Figure S8) and the in-plane radial distribution functions (RDFs) of the DPPC systems (see Figure S7). The change from positive to negative correlation between *S*_chain_ and cholesterol mol % at 300–310 K, and the decrease in translational order with increasing temperature at ≤ 30 mol %, confirm the gradual loss of translational order with increasing temperature and indicates an increase in chain order with rising cholesterol content in the fluid bilayer phase. The slight reduction of the chain order parameter upon increasing cholesterol between 280–300 K occurs close to the *S*_*o*_–*L*_*d*_ transition, where cholesterol can partially disrupt gel-like packing before the lipid ordering trend dominates in the fluid regime.

We also examined how cholesterol influences bilayer thickness. This variable is important for the NEMD simulations discussed below because the temperature gradient across the bilayer and the thermal conductance are expected to vary with bilayer thickness. The bilayer thickness increased with cholesterol content from 10 to 50 mol % for POPC and DLiPC, with DPPC thickness increasing between 10 and 30 mol % and stabilizing above 30 mol % cholesterol composition (see Figure S18). The changes observed in bilayer thickness with cholesterol content are consistent with previous studies.^65–68^

Our findings for DPPC and POPC closely align with both experimental^60,69,70^ and computational^66,71,72^ studies of fluid-phase bilayers under equilibrium conditions (ranging from 310 to 340 K). The decreasing thickness from DPPC to DLiPC at all compositions is expected due to the increasing level of unsaturation. The trend DPPC:CHOL > POPC:CHOL > DLiPC:CHOL reflects the reduction in hydrophobic thickness with increasing unsaturation. DLPC:CHOL can be thicker than DLiPC:CHOL because saturation and cholesterol-induced ordering keep DLPC chains more extended, whereas polyunsaturated DLiPC chains exhibit stronger conformational disorder, reducing thickness.

To set up the NEMD simulations, we used the DPPC melting temperature to select a temperature range, ensuring that both lipid leaflets in each bilayer were above the melting temperature. Temperature gradients were then applied to systems with overall cholesterol mole fractions ranging from 10 to 50 mol %. The magnitude of the thermal gradients was on the order of 10^9^ K m^–1^, which is consistent with previous simulation studies that demonstrated linear response behavior in the thermal transport calculations.^47,56,73^

Figure 2 shows the temperature and density profiles for a 50:50 DPPC:CHOL system. Similar results were obtained for the other bilayer systems studied in this work, as shown in Figures S12–S15. The water density decreases in the hot regions near the edges of the simulation box, consistent with liquid thermal expansion at elevated temperatures. Although the water density at the bilayer center is low, it is not zero (see Figure S16), indicating that the bilayer is permeable to water. This permeability allows water to transfer from the hot to the cold regions, helping to equilibrate the liquid density across the system.

Cholesterol molecules are located closer to the bilayer center than the phospholipid PO_4_ headgroups, reflecting cholesterol’s lower hydrophilicity, which results in its intercalation between lipid tails. The asymmetry in the density maxima of ρ_CHOL_ across the two bilayer leaflets (see Figure 2) indicates a preferential accumulation of cholesterol in cold regions. This suggests that cholesterol, modeled using the coarse-grained MARTINI force field, is thermophobic. This result is consistent with findings from a previous study using an atomistic force field.^47^

Across all systems, the bilayer thermal conductivity generally decreased with increasing overall cholesterol mole fraction (see Figure 3 and Table S4). Additionally, bilayers with higher acyl chain saturation exhibited greater thermal conductivity, indicating that increased lateral packing density facilitates heat transfer. This is supported by the larger thermal conductivity observed in the DBPC and DXPC bilayer. Analysis of the density profiles reveals a density enhancement in the middle of the DBPC and DXPC bilayers (Figures S14 and S15), rather than the depletion observed in e.g. DPPC (Figure 2) and POPC (see maximum in the interior of the bilayer in the HC_Chain density profiles shown Figures S12 and S13). These results indicate some degree of interdigitation in the DBPC and DXPC bilayers. This is confirmed in the snapshots shown in Figures S14 and S15 (see snapshots shown as embedded insets/overlays in the corresponding figures). This structural feature may contribute to the observed increase in thermal conductivity. The thermal conductance (see Figure 3-top) generally follows the cholesterol-induced increase in bilayer thickness (see Figure S18), with hydrocarbon chain straightening accompanying rising cholesterol content. A comparable decrease in thermal conductance is observed in bilayers with extended saturated hydrocarbon chain lengths (DLPC to DXPC) at 50 mol % cholesterol (Figure 3, top), paralleling the thickness-dependent trend seen in cholesterol-varying systems. Overall, the thermal conductances obtained here, 20–30 MW/(K m^2^), are similar to interfacial thermal conductances found in experimental studies of hydrophobic interfaces.^74^

**Figure 3:**
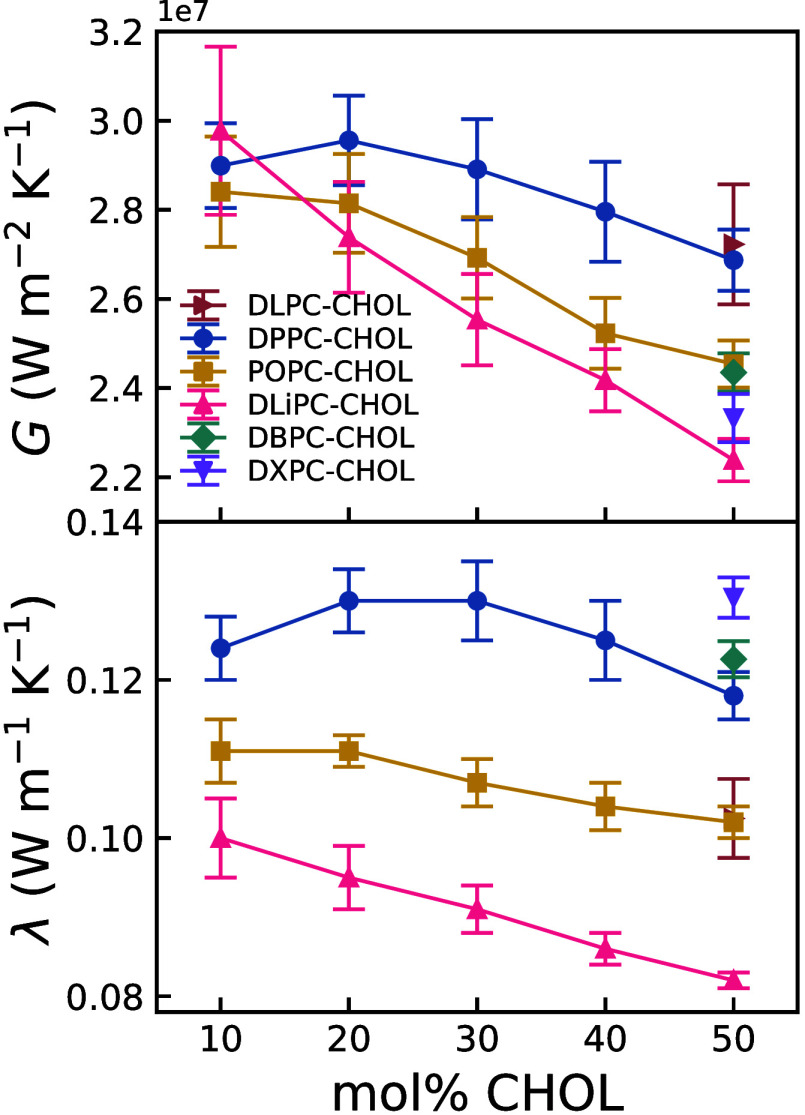
Thermal conductance (top) and thermal conductivity (bottom) of lipid bilayers as a function of cholesterol mole fraction for DPPC, POPC and DLiPC. For the remaining lipid systems (DLPC, DBPC and DXPC), we show results for a single cholesterol mole fraction, 50 mol %. The overall cholesterol mole fraction (mol %) is defined as *N*_*CHOL*_/(*N*_*CHOL*_ + *N*_*P*_) × 100, where *N*_*CHOL*_ and *N*_*P*_ are the total number of cholesterol and phospholipid molecules in the bilayer, respectively. The uncertainty represents the standard deviation of the mean of five replicas.

Under comparable local temperature differences, our simulations yielded λ = 0.124 ± 0.004 W/(K m) for 90:10 DPPC:CHOL, λ = 0.111 ± 0.004 W/(K m) for 90:10 POPC:CHOL, and λ = 0.100 ± 0.005 W/(K m) for 90:10 DLiPC:CHOL, with all systems maintained at an average temperature of ∼ 347 K. The MARTINI 3 bilayers exhibit thermal conductivities in the range 0.08–0.14 W /(K m) across the compositions investigated. These values are lower than those typically reported by atomistic NEMD studies for fluid-phase phosphatidylcholine bilayers (0.25–0.32 W /(K m) for DPPC/DLPC/SMPC and up to 0.42–0.58 W /(K m) for DPPC),^43,44,75^ consistent with the expectation that coarse-graining removes intramolecular vibrational/conformational channels that can enhance heat transport in all-atom force fields.

Direct experimental benchmarks for isolated freestanding bilayers remain limited, but experimental studies of mammalian lipid-rich structures report a thermal conductivity of 0.21–0.31 W /(K m), noting that extrapolations to 100% lipid composition in those works yield values of order 0.1–0.15 W/(K m).^76^

We note that direct experimental benchmarks for single lipid bilayers are limited, so our comparison primarily emphasizes consistency with prior simulation ranges and the physical trends across composition. Taken together, these comparisons indicate that while absolute conductivities are model dependent, the coarse-grained simulations are well suited to capture composition-dependent trends, including the systematic reduction of the thermal conductivity with increasing cholesterol content and the higher heat transport efficiency observed in more saturated bilayers. In the present work, we interpret the MARTINI 3 results primarily in terms of robust, composition-dependent trends (e.g., changes with cholesterol loading and lipid saturation) rather than as quantitatively predictive absolute conductivities.

To investigate how the temperature gradient influences bilayer asymmetry, we calculated the time-dependent fraction of cholesterol in the hot and cold leaflets. The time-dependent cholesterol fraction is defined as

$$x_{CHOL,\alpha }\left(t\right)=\frac{N_{CHOL,\alpha }\left(t\right)}{N_{CHOL,T}}$$ 3

where *N*_*CHOL*,α_(*t*) is the number of cholesterol molecules in leaflet α = (“h” for the hot leaflets or “c” for the cold leaflets) at time *t*, and *N*_*CHOL*,*T*_ is the total number of cholesterol molecules in a bilayer. The time scale for phospholipid flip-flop is on the order of minutes to hours.^14,16^ Since no phospholipid flip-flop events occur within our simulation time scales of 1–2 μs, the number of phospholipids per leaflet is constant throughout the simulation, and they were not included in the calculation of the cholesterol fraction. Consequently, the cholesterol fraction is set to 0.5 at the beginning of the simulation, when the membrane is fully symmetric. As time progresses, the cholesterol fraction increases in the cold leaflet and decreases by the same amount in the hot leaflet.

Figure 4 shows the progression toward a steady state cholesterol distribution in a DPPC bilayer with a 50:50 cholesterol-to-phospholipid ratio. The membrane is initially symmetric, but over time, cholesterol becomes enriched in the colder region and depleted in the hotter region. The composition reaches a well-defined steady state within approximately 100 ns. Thereafter, the cholesterol fraction fluctuates around this steady-state value throughout the 1 μs simulations.

**Figure 4:**
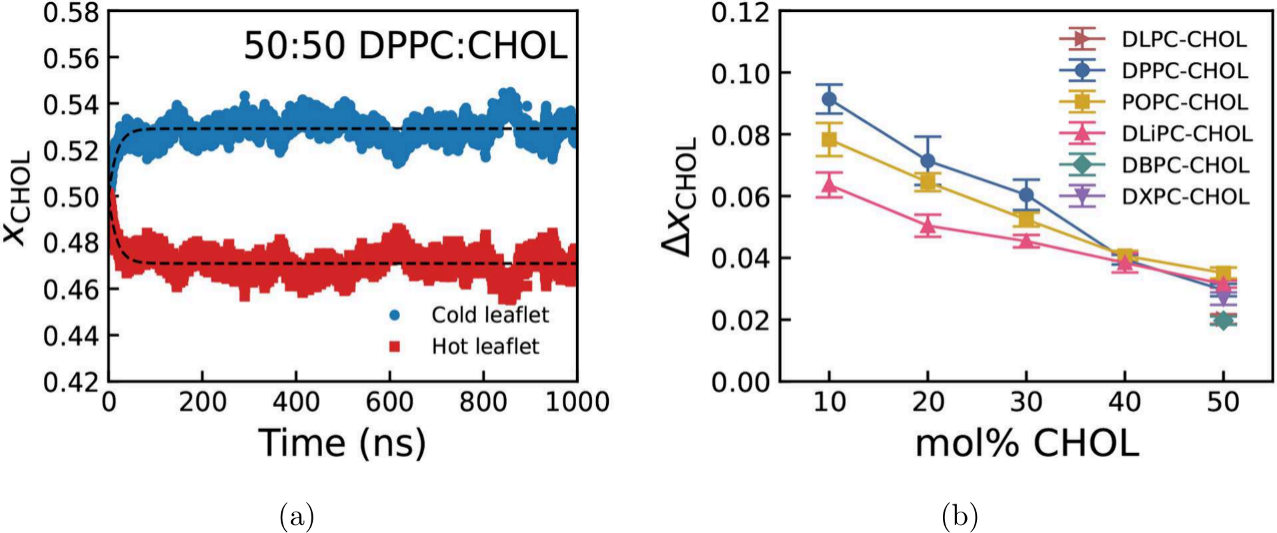
(a) Fraction of cholesterol in the hot (red) and cold (blue) leaflets over time. The thermal gradient is ∇*T*_*bilayer*_ = 4.3 K/m. The dashed lines represent fits to the data using eq 4. (b) Change in steady-state cholesterol fraction as a function of the total cholesterol mol %. For the lipid systems DLPC, DBPC, and DXPC, data are shown for a single cholesterol fraction of 50%. Δ*x*_*CHOL*_ = *x*_*CHOL*,*c*_^*s*^ – *x*_*CHOL*,*c*_(0), where *x*_*CHOL*,*c*_^*s*^ and *x*_*CHOL*,*c*_(0) are the steady-state and initial cholesterol fractions in the cold leaflet, respectively. Note that the results are identical for the hot leaflet, but Δ*x*_*CHOL*_ is negative. The uncertainty represents the standard deviation of the mean of five replicas.

Our results indicate that cholesterol exhibits thermophobic behavior, consistent with earlier simulations using an atomistic model of a 50:50 DPPC:CHOL bilayer.^47^ The accumulation of cholesterol in colder regions reflects its greater affinity for ordered lipid tails. Indeed, an analysis of the order parameter for the two leaflets confirms this trend: the average lipid order parameter, *S*_*chain*_, which quantifies the ordering of the coarse-grained chains, is higher in the cold leaflet (see SI for details and Figure S10). Our results indicate that cholesterol thermophobicity reflects its equilibrium preference for more ordered lipid environments, which are favored at lower temperatures. Under a temperature gradient, this yields a free-energy difference that drives cholesterol enrichment on the cold side. This provides a microscopic mechanism to explain the thermophobicity of cholesterol.

Figure 4b shows the cholesterol asymmetry, Δ*x*_*CHOL*_ = *x*_*CHOL*,*c*_^*s*^ – *x*_*CHOL*_(0), where *x*_*CHOL*,*c*_(0) = 0.5, as fraction of cholesterol in the cold leaflets over total number of cholesterol at time 0. The accumulation of cholesterol in the cold region decreases with the cholesterol content of the bilayer. This trend is most pronounced for fully saturated DPPC and diminishes with increasing levels of PC unsaturation. The impact of unsaturation is clearly visible at low cholesterol content, ∼10 mol % corresponding to the *L*_*d*_ phase. At 40 mol % or higher cholesterol content, corresponding to *L*_*o*_ phases, the translocation of cholesterol from the hot to the cold region becomes independent of the PC saturation level. Given cholesterol’s higher affinity for ordered environments, our results indicate that in the *L*_*d*_ phase, the influence of thermal-gradient-induced asymmetry on cholesterol partitioning is most pronounced in saturated PC bilayers, owing to their more efficient lipid packing.

We performed additional simulations across a range of thermal gradients (see Figures S23, S24, S25, S26, and S27). In all cases, and for all lipid bilayers investigated, cholesterol exhibits thermophobic behavior. The asymmetry increases proportionally to the gradient, supporting a linear response in cholesterol partitioning under thermal stress.

To quantify the translocation of cholesterol across the bilayer, we used the kinetic model^47^ (see the SI)­

$$x_{CHOL,\alpha }\left(t\right)=x_{CHOL,\alpha }\left(0\right)+\left(x_{CHOL,\alpha }^{s}-x_{CHOL,\alpha }\left(0\right)\right)\left(1-\exp\left(-t/\tau \right)\right)$$ 4

where *x*_*CHOL*,α_(0) = 0.5 is the initial fraction of cholesterol (see Equation 3) in leaflet α, *x*_*CHOL*,α_^*s*^ is the steady-state fraction, and the time constant τ = *k* ^–1^ = (*k*_*ch*_ + *k*_*hc*_)^−1^, where *k* is the rate constant. This kinetic model assumes two rate constants that quantify the dynamics of cholesterol movement from cold to hot (*k*_*ch*_) or from hot to cold (*k*_*hc*_). Advancing the discussion below, the translocation pathway of cholesterol may feature (depending on the bilayer composition) a local minimum when cholesterol molecules are located in the bilayer midplane. However, this minimum involves activation barriers that are much smaller than those associated with movement from the equilibrium position in the leaflet. Hence, the time/rate constant defined above should provide a good approximation for our analysis. Indeed, the kinetic model described in Equation 4 fits the simulation data remarkably well. For the 50:50 DPPC:CHOL bilayer shown in Figure 4a, the time constant is τ = *k* ^–1^ = 16.1 ns, indicating that the system reaches a stationary state very rapidly.

Figure 5 shows the time constants for the different bilayers investigated in this work. For clarity, we discuss translocation primarily in terms of the relaxation time τ. In all cases, the steady-state fraction is reached in less than 30 ns in the coarse-grained models. While we do not observe a clear trend in the time constant τ with cholesterol content, the impact of PC saturation level on cholesterol translocation dynamics is significant. This finding suggests that the rate constant, *k* = *k*_*ch*_ + *k*_*hc*_ decrease as the degree of PC saturation increases. Consequently, cholesterol translocation across the bilayer slows down, likely due to an altered energy landscape characterized by higher activation barriers in bilayers with more saturated PC chains.

**Figure 5:**
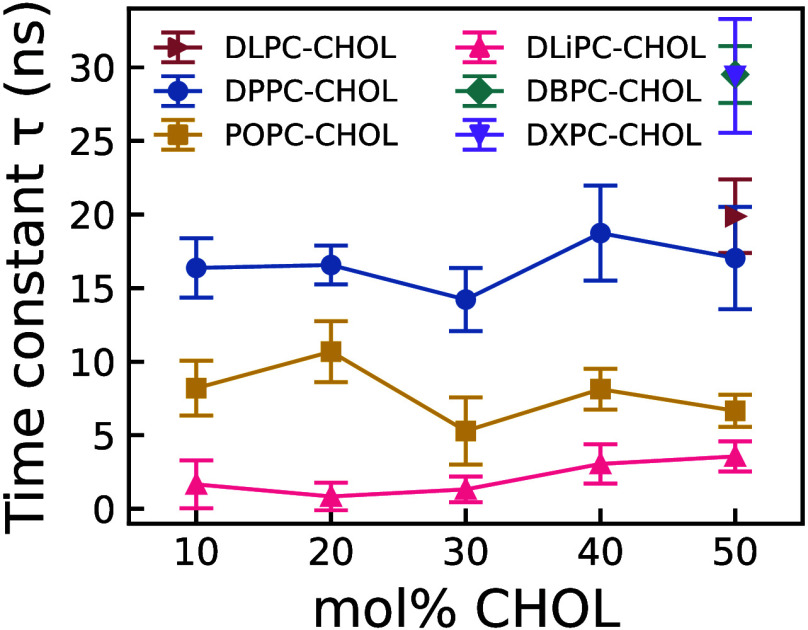
Time constant for cholesterol translocation across the bilayer under a thermal gradient, shown as a function of cholesterol content for DPPC:CHOL, POPC:CHOL, and DLiPC:CHOL (10–50 mol % CHOL). For DLPC:CHOL, DBPC:CHOL, and DXPC:CHOL, data are shown for a single cholesterol composition (50 mol % CHOL). Refer to the caption of Figure 3 for the definition of cholesterol content. Error bars denote the standard deviation of the mean of τ obtained from five independent simulation replicas. The reported τ values correspond to the exponential relaxation time of a two-state model and relate to the microscopic rates via τ = 1/(*k*_*ch*_ + *k*_*hc*_). See main text.

To analyze the translocation pathway of cholesterol molecules in the thermal field, we computed the combined probability distribution *P*(*z*, θ). This distribution quantifies the likelihood of finding the cholesterol headgroup (ROH) at a specific height, *z*, within the bilayer, where *z* = 0 corresponds to the bilayer center. The second parameter, the tilt angle, θ, measures the orientation of cholesterol relative to the bilayer normal. The definition of the tilt angle is provided in Figure S3 in the Supporting Information.

The free energy surface of a DPPC:CHOL (50:50) bilayer is shown in Figure 6, represented as $-\overset{\sim }{1n\,\;\;\;P(z},\theta )\;$ and projected onto the *z*, θ plane. The heat map closely resembles those obtained from the atomistic model (see ref 47). For the DPPC:CHOL bilayer, we identify five distinct free-energy minima. The two most populated states, A and B, correspond to cholesterol with its headgroup positioned slightly below the water–membrane interface, exhibiting tilt angles of approximately 13° and 20° toward the normal of the cold and hot leaflets, respectively. These tilt angles agree well with previous studies, which reported values of 10° and 14°,^47^ and 19.7°^77^ using atomistic force fields, as well as 16°–19° observed experimentally.^78^

**Figure 6:**
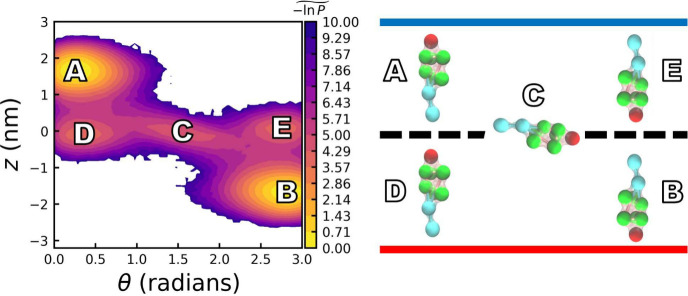
(Left panel) Heatmap of the normalized negative logarithm of the population of states shown as $\left(-\overset{\sim }{1n\,\;\;\;P(z},\theta )\;=-\ln P\left(z,\theta \right)/P_{ref}\right)$ , determined by the position of the ROH group (indicated by the red bead in the right panel) relative to the bilayer center (*z* = 0) and the cholesterol tilt angle (θ), of the DPPC:CHOL 50:50 system. The five equilibrium states of cholesterol, corresponding to minima in the free-energy surface, are labeled A–E. The reference probability *P*_*ref*_ ≡ max_*z*,θ_ *P*(*z*, θ) occurs at state A. The color scale is shifted so that the minimum is 0 and clipped at 10 (lower values indicate lower free energy and greater stability). (Right panel) The cartoon illustrates the equilibrium states of cholesterol (A–E). The dashed line represents the midbilayer plane, while the red and blue horizontal lines indicate the positions of the hot and cold leaflets, respectively. The free energy surface, $\Delta F\left(z,\theta \right)/\left(k_{B}T\left(z\right)\right)=-\ln\left(P\left(z,\theta \right)/P{\left(z,\theta \right)}_{\min}\right)=-\overset{\sim }{1n\,\;\;\;P(z},\theta )\;$ .

A secondary free-energy minimum (state C) occurs at the bilayer midplane, where cholesterol adopts an orientation parallel to the membrane plane. This metastable state has a lower free energy than states D and E, reflecting its intermediate position along the cholesterol flip-flop pathway. The least populated states, D and E, correspond to configurations in which the hydroxyl group is embedded near the bilayer center and the sterol tail points toward the aqueous phase. The right panel of Figure 6 illustrates the five minima identified in the free-energy landscape.

Figure 7 shows heatmaps for bilayers with varying phosphatidylcholine (PC) chain saturation and different hydrocarbon chain lengths. Both the degree of saturation and chain length significantly influence the free-energy landscape. A high level of saturation promotes the emergence of distinct metastable states D and E, observed in the 50:50 DPPC:CHOL, 50:50 DBPC:CHOL, and 50:50 DXPC:CHOL systems. The regions between these minima are sparsely populated, consistent with a high energy barrier and strong leaflet ordering that restrict cholesterol mobility. In contrast, the heat maps of 50:50 DLPC:CHOL, 50:50 POPC:CHOL, and 50:50 DLiPC:CHOL bilayers are considerably simpler, featuring two main minima corresponding to states A and B (depicted in the right panel of Figure 6), along with a horizontally aligned metastable state near the bilayer center. DLPC stands out among saturated lipids: its short chains form a thin bilayer, reducing hydrophobic thickness and lowering the energetic penalty for cholesterol movement, which explains its simpler landscape. Overall, the heat maps of the two unsaturated lipid systems (DLiPC and POPC) show a broader spread and indicate reduced free-energy barriers for cholesterol translocation. This suggests that the lateral chain disorder induced by double C = C bonds facilitates cholesterol movement from hot to cold regions and allows cholesterol molecules to explore intermediate positions inside the bilayer more readily.

**Figure 7:**
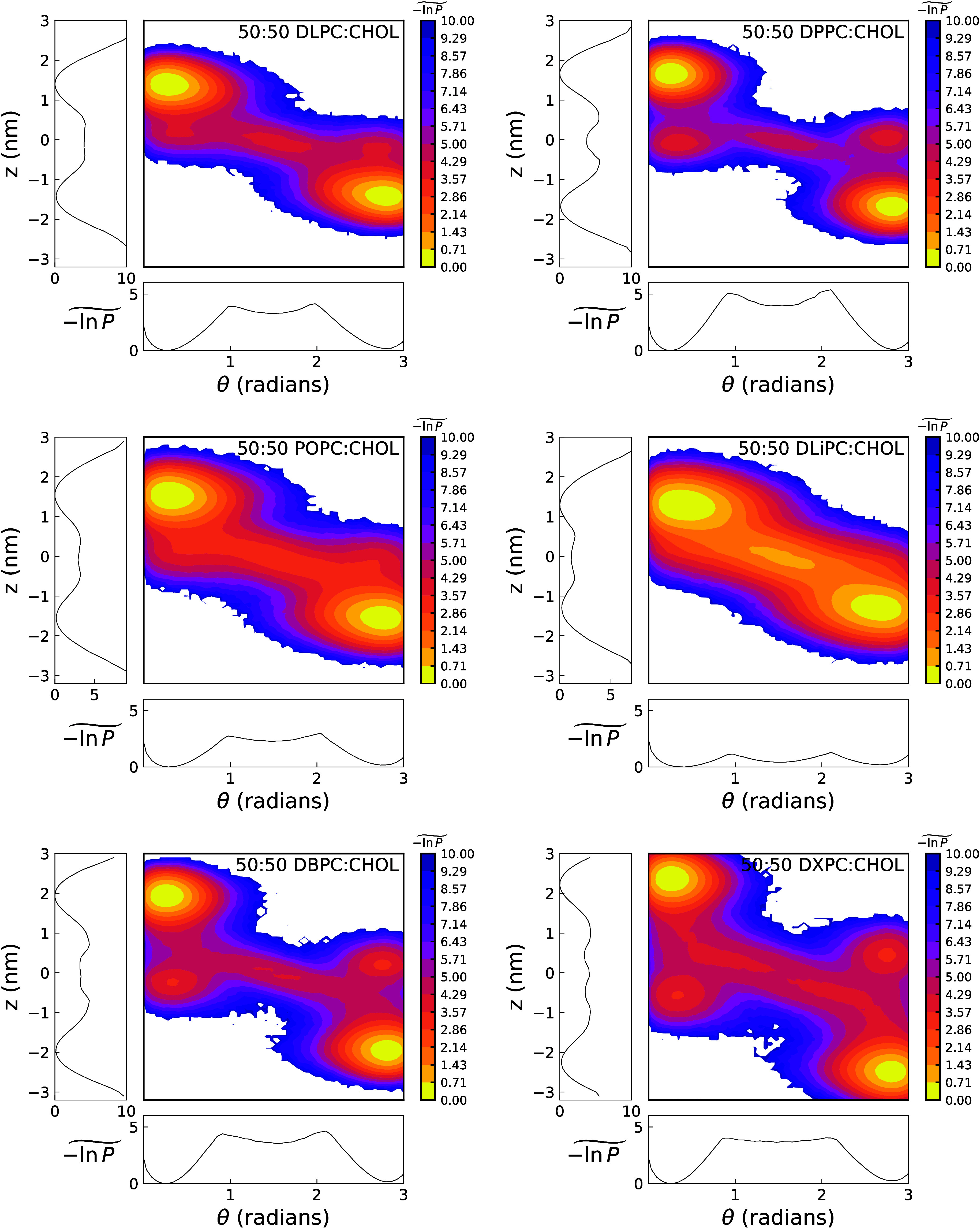
Free-energy landscapes of bilayers containing 50 mol % of cholesterol. The additional panels on the left and bottom of each plot show $-\overset{\sim }{1n\,\;\;\;P(z},\theta )\;$ projected along the *z* (left) or θ (bottom) order parameters. The projection is performed by selecting, for a given value of the remaining coordinate, the minimum of – ln *P*(*z*, θ) over the projected variable, yielding the lowest free-energy cost associated with the most favorable configuration.

In DPPC:CHOL systems with varying cholesterol content (Figure S28), the free-energy landscape of the 90:10 DPPC:CHOL system, corresponding to the *L*_*d*_ phase, is simplified to three main minima: states A, C, and B, as illustrated in Figure 6. Metastable states D and E appear at 20 and 30 mol % cholesterol, but their stability increases in the *L*_*o*_ phase region when the overall cholesterol mole fraction reaches 40 mol % or higher. Our results indicate that PC chain saturation and high cholesterol content in the *L*_*o*_ phase, where cholesterol–cholesterol interactions are significant, promote the formation of metastable states in which cholesterol orientations are energetically unfavorable with respect to the orientation where the OH points toward the aqueous phase.

Figure 8 compares the free-energy profiles for bilayers with varying saturation levels and chain lengths, focusing on a 50:50 composition corresponding to the *L*_*o*_ phase. The results show that free-energy barriers increase with the saturation level of the PC chain, indicating that cholesterol translocation requires greater activation energy in fully saturated DPPC bilayers. This finding is consistent with the data presented in Figure 5, which shows that the characteristic time for cholesterol translocation rises with increasing PC chain saturation. DLiPC and POPC exhibit the lowest and second-lowest activation barriers, consistent with the shorter time constants (faster rates) observed for these bilayers, indicating that translocation dynamics accelerate with increasing unsaturation. This observation aligns with the structural properties of the bilayers: longer saturated chains increase overall bilayer order and thickness, stabilizing cholesterol within a leaflet; however, as the lipid chain increases, the bilayer center becomes increasingly dominated by flexible methyl termini.^79^

**Figure 8:**
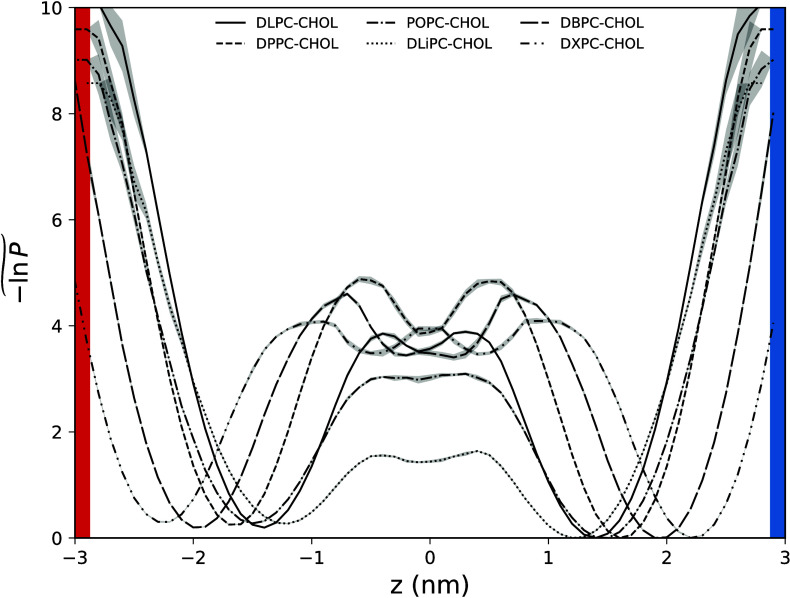
One-dimensional free-energy profiles for cholesterol position as a function of its position *z* relative to the bilayer midplane *z* = 0. The free energy is defined as $-\overset{\sim }{1n\,\;\;\;P}=\underset{\theta }{\min}\left\{-\ln\left[P\left(z,\theta \right)/P_{\mathrm{ref}}\right]\right\}$ , i.e, the minimum over tilt angles θ. Profiles are shifted by a constant so that the global minimum over (*z*, θ) is zero. This minimum lies in the cold leaflet (blue vertical band). All data correspond to bilayers with 50 mol % cholesterol. *P*(*z*, θ) histograms. Shaded envelopes indicate the statistical uncertainty (±1 standard deviation) across five independent replicas. The *y*-axis is truncated at 10 for readability. See Figure S30 for a comparison of the free-energy profiles obtained from different replicas.

The termini of longer saturated hydrocarbon chains (see Figure S15) create a high-density region in the bilayer midplane rather than a gap, suggesting a tendency toward interdigitation. This explains the lower probability of cholesterol occupying that region and the absence of a metastable minimum in the free-energy surface (see DXPC:CHOL system in Figure 7 and the maximum in the free-energy profile of DXPC at *z* = 0, in Figure 8). DLPC lipids form the thinnest bilayer and the steepest local temperature gradient (Table S3). The energy barrier for DLPC is similar to that of other saturated lipids, which is reflected in comparable translocation time constants (see Figure 5).

Overall, these observations indicate that cholesterol translocation under a thermal gradient is governed by the interplay of multiple factors: phospholipid saturation level, bilayer thickness, lipid interdigitation, and packing efficiency.

We have demonstrated that thermal fields induce asymmetry in cholesterol distribution across lipid bilayers. Our results highlight that this asymmetry depends on the bilayer saturation level and hydrocarbon chain length. We now quantify the asymmetry using the Soret coefficient:^80^

$$S_{T}=-\frac{1}{x_{CHOL}^{o}x_{PC}^{o}}{\left(\frac{\Delta x_{CHOL}^{\prime }}{\Delta T}\right)}_{s}$$ 5

This is a finite difference form of the Soret equation, where *x*_*CHOL*_^*o*^ and *x*_*PC*_^*o*^ represent the overall mole fractions of cholesterol and phosphatidylcholine, respectively. Δ*x* *′*_*CHOL*_ and Δ*T* are defined in the stationary state, *s*: Δ*x* *′*_*CHOL*_ = *x* *′*_*CHOL*,*h*_ – *x* *′*_*CHOL*,*c*_ denotes the difference in steady-state mole fractions of cholesterol between the hot (α = *h*) and cold (α = *c*) leaflets, while Δ*T* = *T*_*h*_ – *T*_*c*_ is the temperature difference between the leaflets. The steady-state mole fraction of cholesterol in each leaflet, *x* *′*_*CHOL*,α_, is given by

$$x_{CHOL,\alpha }^{\prime }=\frac{N_{CHOL,\alpha }}{N_{CHOL,\alpha }+N_{PC,\alpha }}$$ 6

The Soret coefficients (see Figure 9) have values typical of aqueous solutions^81^ and exhibit a systematic decrease with increasing cholesterol mol %. This suggests that the Soret effect is more pronounced in bilayers with low cholesterol content, with a general trend toward thermodiffusion enhancement as PC saturation increases. At high cholesterol content (50 mol %), characteristic of the *L*_*o*_ phase, the Soret coefficient becomes insensitive to PC saturation. The values we obtain for the DPPC:CHOL 50:50 composition, (0.262 ± 0.030) × 10^–2^ K^–1^, are comparable to those reported in previous atomistic simulations of 50:50 DPPC:CHOL bilayers.^47^

**Figure 9:**
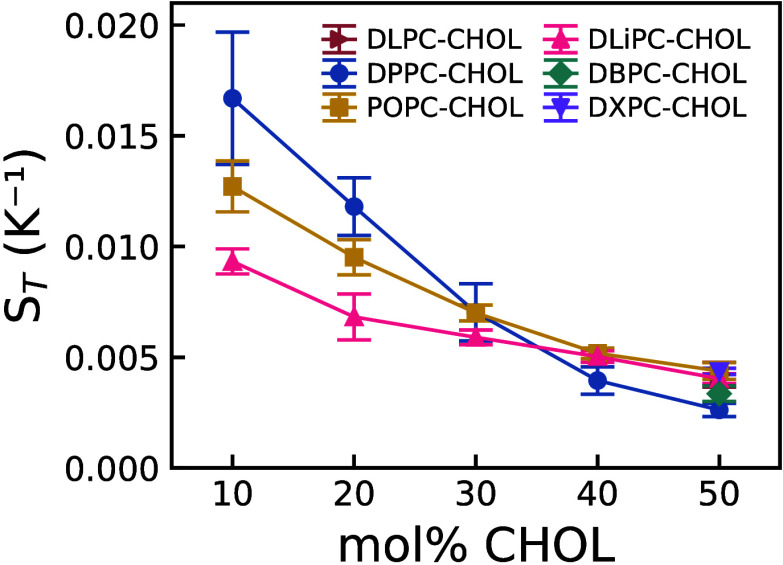
Soret coefficient as a function of cholesterol mole fraction for lipid bilayers with varying degrees of PC saturation. Note that, for DLPC, DBPC, and DXPC, data are shown for a single cholesterol mole fraction of 50 mol %. Error bars represent uncertainties obtained from the standard deviation of the steady-state cholesterol mole fraction difference and the uncertainty in the applied temperature difference.

Our analysis of the Soret coefficient provides insight into how local heat fluxes, potentially arising from metabolic activity or external stimuli, can modulate lipid membrane asymmetry and organization.These findings have implications beyond biophysics, including thermal therapies and nanoscale heat management, where temperature gradients may regulate membrane structure and function.

## Conclusion

Using nonequilibrium molecular dynamics with the MARTINI 3 coarse-grained force field, we quantified cholesterol thermodiffusion and heat transport in phosphatidylcholine bilayers exposed to transverse thermal gradients. Cholesterol is consistently thermophobic, enriched in the colder leaflet across lipid compositions and cholesterol mole fractions (10–50 mol %), in both liquid-disordered and liquid-ordered phases. This asymmetry is strongest at low cholesterol content and increases with phospholipid saturation, as reflected by larger Soret coefficients, whereas at 50 mol % cholesterol the Soret response becomes weak and largely insensitive to saturation. Notably, the Soret coefficients predicted by coarse-grained models align with those obtained in previous atomistic simulations, supporting the generality of our observations. We emphasize that this agreement for the benchmark DPPC:CHOL system supports the use of MARTINI 3 to capture the direction of cholesterol thermodiffusion and the associated composition-dependent trends driven by lipid packing and chain order. At the same time, absolute thermal conductivities are expected to be more model dependent in coarse-grained descriptions because intramolecular vibrational and conformational contributions to heat transport are reduced. Accordingly, we interpret our thermal-conductivity results primarily in terms of robust trends with cholesterol loading and lipid saturation rather than as quantitatively predictive absolute values. These predictions provide guidance for future analyses using atomistic models and, potentially, experiments.

Cholesterol translocation kinetics depend strongly on bilayer composition. Saturated bilayers exhibit slower flip-flop and higher activation barriers than unsaturated membranes. Thermal transport follows complementary trends, with higher thermal conductivity in saturated bilayers, and a systematic reduction of thermal conductivity upon increasing cholesterol content.

Within linear response, the thermodiffusive enrichment scales with the temperature difference between cold and hot regions in the bilayer, Δ*T*, and may be small for small Δ*T*, e.g. ∼1 K, but could be amplified in other (composition-dependent) membrane mixtures and under strong, localized thermal stresses, where modest local changes in sterol content may impact membrane order and lateral organization. Future extensions of this work might include nonequilibrium simulations of other membrane compositions and curved membrane geometries (e.g., vesicles) to quantify how curvature–composition coupling and leaflet stress asymmetry modulate cholesterol thermodiffusion.

Overall, we have shown that lipid saturation and cholesterol content jointly control mass and heat transport in membranes under thermal stress. These results are relevant for understanding how local thermal stress can reshape membrane composition and transport properties.

## Supplementary Material



## Notes

The Supporting Information provides full simulation protocols and analysis details, including the NEMD setup, system sizes and compositions (Tables S1–S2), temperature/density profiles and bilayer parameters (Table S3; Figures S12–S18), heat-flux/thermal-transport results (Table S4; Figure S19), cholesterol flip-flop kinetics and Soret-coefficient calculations (Table S5; Figures S20–S27), free-energy surfaces and replica comparisons (Figures S28–S30), as well as energy-conservation tests, derivation of the kinetic model and uncertainty propagation methods. (PDF)
