# Supplementary material for: Phospholipid Saturation Modulates Cholesterol Partitioning and Heat Transport in Lipid Bilayers under Thermal Gradients
Source: Langmuir. 2026 May 21;42(21):14635–46. doi: 10.1021/acs.langmuir.5c06531 (PMC13235634; doi:10.1021/acs.langmuir.5c06531)
Supplement: Supplementary file 1 [file la5c06531_si_001.pdf]

Supporting Information: Phospholipid Saturation Modulates Cholesterol Partitioning and Heat Transport in Lipid Bilayers under Thermal Gradients

Zhibo Deng, Mona W. Qiu, Fionn Carman, John M. Seddon, and Fernando Bresme*

Department of Chemistry, Imperial College London, Molecular Sciences Research Hub, 80 Wood Lane, London, W12 0BZ, UK.

E-mail: f.bresme@imperial.ac.uk

Phone: +44(0)207 594 5886

Simulation Details

Equilibrium simulations were performed using the velocity-rescale thermostat[S1] with a time constant of 1.0 ps. Pressure was maintained at 1 bar using the C-rescale[S2] and Parrinello-rahman[S3] barostats before and after duplication of the single bilayer with a time constant of 5.0 ps. The compressibility was set to 3.0×10^{-4} bar^{-1}, and separate barostats were applied in the directions perpendicular (z) and parallel (x, y) to the bilayer plane. The Berendsen barostat was used only during the initial energy equilibration stages, to rapidly relax large pressure mismatches and remove residual stresses from the starting configuration. It was not used during pre-equilibration or equilibration. The pre-equilibration for each single bilayer

lasted ∼100 ns at 300 K and consisted of five stages, each with an increasing timestep and a decreasing number of steps: 5×10^5 steps at 2 fs, 2×10^5 steps at 5 fs, 10^5 steps at 10 fs, 5×10^4 steps at 15 fs, and 5×10^6 steps at 20 fs. Convergence of the pre-equilibrated bilayer was assessed and shown in Figure S1, with the absence of systematic drift in the area per lipid, and the plateauing of their cumulative means after approximately 25 ns of the final 100 ns segment. Following duplication of the simulation box along the z direction to obtain the two bilayers system, further equilibration at the mean temperature $T_{\text{avg}} = (T_{\text{hot}} + T_{\text{cold}})/2$, where T_{hot} and T_{cold} are the temperature for the hot and cold thermostats, was performed for 10^7 steps using a 20 fs integration timestep (200 ns).

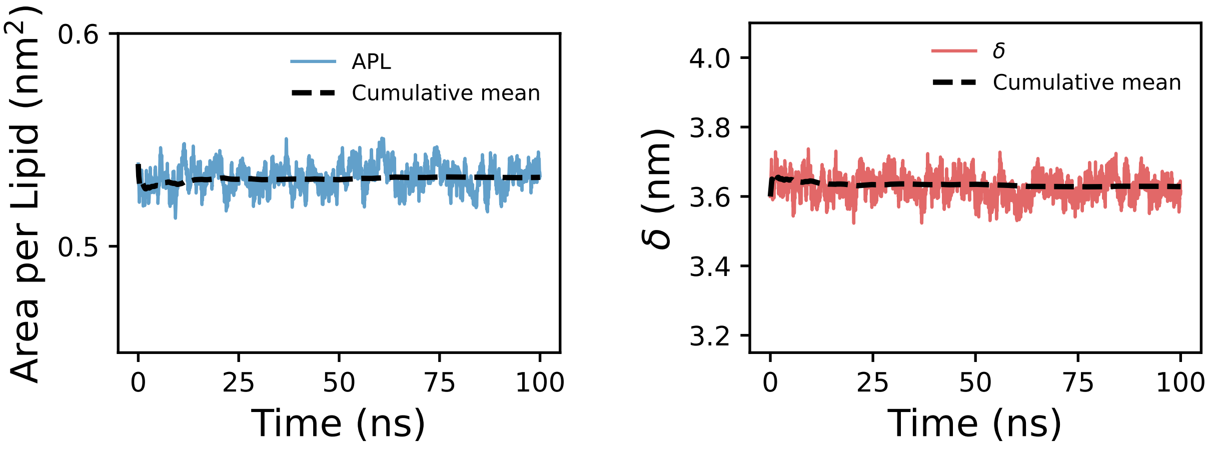

(a) (b)

Figure S1: Time evolution of (a) area per lipid (APL) (average area of the bilayer divided by the number of lipids per leaflet) and (b) bilayer thickness δ as a function of time during the last segment of pre-equilibration (100 ns) of the single DLiPC:CHOL 50:50 bilayer system. Solid lines show instantaneous values, while dashed lines indicate the cumulative (expanding) mean, computed as $\overline{x}(t_n) = \frac{1}{n}\sum_{i=1}^{n} x(t_i)$, where $x(t_i)$ is the observable sampled at time t_i and n is the number of samples accumulated up to t_n.

Nonbonded interactions were calculated using a 1.1 nm cutoff and the potential-shift-Verlet modifier to avoid force discontinuities at the cutoff radius.[S4] The relative dielectric permittivity was set to $\varepsilon_r = 15$ to account for explicit screening in MARTINI water.[S5] Electrostatic interactions were computed using the reaction-field method.[S6]

For nonequilibrium simulations (Figure S2), we updated the neighbor list and removed

the center-of-mass motion for the whole system. Our tests indicated that using longer update intervals led to poor energy conservation and significant energy drift. See energy conservation discussion below for more information.

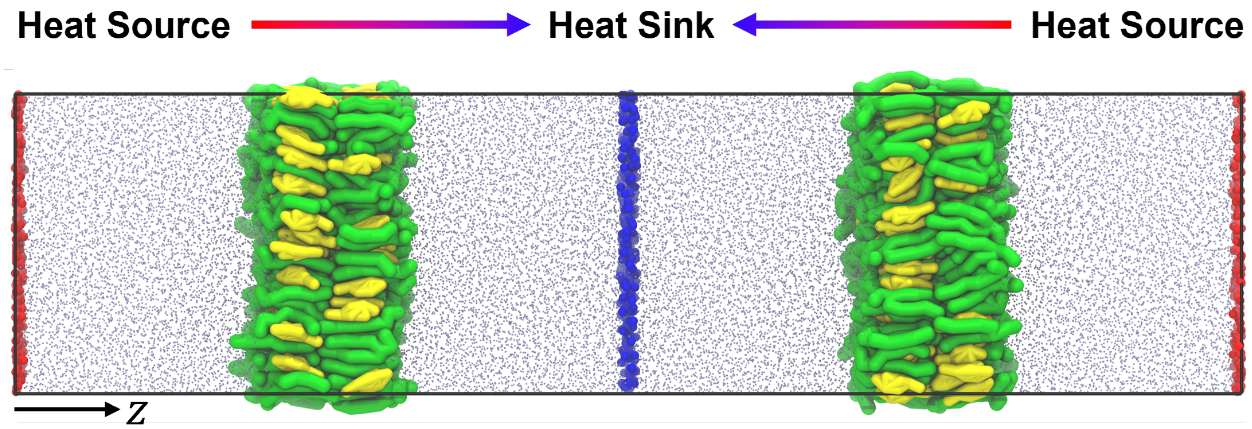

Figure S2: Snapshot of a representative 50:50 DPPC:CHOL configuration used in NEMD simulations in this work, visualized by VMD[S7] and MartiniGlass.[S8] Cholesterol is represented in yellow.

Table S1 and S2 lists the systems and simulation summary investigated in this work.

Table S1: System dimensions and atom counts for lipid bilayers with varying cholesterol content. L_ξ is the box length in direction ξ in nm.

**Lipids**	**Composition**	**Atoms**	$\mathbf{L_x}$	$\mathbf{L_y}$	$\mathbf{L_z}$
DPPC:CHOL	100:0	24035	12.748	12.748	26.760
	90:10	32733	11.628	11.628	38.885
	80:20	32126	10.992	10.992	41.296
	70:30	31100	10.616	10.616	42.625
	60:40	29716	10.364	10.364	42.583
	50:50	28648	10.226	10.226	41.823
POPC:CHOL	100:0	26444	13.323	13.323	26.318
	90:10	36384	12.337	12.337	36.691
	80:20	34750	11.877	11.877	37.741
	70:30	33406	11.423	11.423	38.964
	60:40	31818	11.003	11.003	40.019
	50:50	30070	10.680	10.680	39.966
DLiPC:CHOL	100:0	29586	14.364	14.364	24.581
	90:10	40520	13.338	13.338	34.273
	80:20	38602	12.929	12.929	34.693
	70:30	36644	12.390	12.390	35.817
	60:40	34616	12.041	12.041	35.750
	50:50	32450	11.465	11.465	37.004
DLPC:CHOL	50:50	29852	10.481	10.481	40.247
DBPC:CHOL	50:50	29769	10.191	10.191	44.505
DXPC:CHOL	50:50	28682	10.163	10.163	44.584

Table S2: Summary of simulation settings and protocols. For entries labeled "300–425 (5 gradients)", the imposed temperature pairs were $T_{\mathrm{cold}} = 300$ K and $T_{\mathrm{hot}} \in \{325, 350, 375, 400, 425\}$ K (i.e., 300–325, 300–350, 300–375, 300–400, and 300–425 K).

System	CHOL (mol%)	Temperature (K)	Pre-eq.	Eq.	Prod.	Reps.
Steady-temperature and composition-dependent simulations						
DLPC–CHOL	50	T_{cold}–T_{hot}	103.75 ns	200 ns	2 μs	5
DPPC–CHOL	50	T_{cold}–T_{hot}	103.75 ns	200 ns	2 μs	5
DBPC–CHOL	50	T_{cold}–T_{hot}	103.75 ns	200 ns	2 μs	5
DXPC–CHOL	50	T_{cold}–T_{hot}	103.75 ns	200 ns	2 μs	5
DPPC–CHOL	10–50	300–400 K	103.75 ns	200 ns	1 μs	5
POPC–CHOL	10–50	300–400 K	103.75 ns	200 ns	1 μs	5
DLiPC–CHOL	10–50	300–400 K	103.75 ns	200 ns	1 μs	5
Temperature-ramping simulations for phase-transition configurations						
DPPC–CHOL	0–50	280 $\rightarrow$ 340 $\rightarrow$ 280 K	103.75 ns	N/A	300 ns	10
POPC–CHOL	0–50	265 $\rightarrow$ 335 $\rightarrow$ 265 K	103.75 ns	N/A	350 ns	10
Equilibrium simulations for phase analysis, $T_{\mathrm{P}} \in \{280, 290, 300, 310, 320, 330, 340\}$ K						
DPPC–CHOL	0–50	T_{P}	4.75 ns	N/A	50 ns	10

For the investigation of phase transitions in pure bilayers, we performed simulations using temperature annealing, with temperatures ranging from 280-340 K and with a heating and cooling rate of 0.4 K/ns. For DPPC:CHOL systems, we selected initial configurations for the annealing trajectories, and an additional set of equilibrium simulations was used to compute radial distribution functions, chain order parameters, and the area per lipid. from 280-340 K (inclusive) in 10 K increments. These equilibrium trajectories were obtained at constant temperature and pressure (1 bar) and lasted 50 ns each. We performed analyses across 10 replicas to obtain averages and standard deviations.

Analysis of the simulation data

Bilayer structure

Lipid molecules were assigned to the upper and lower leaflets using the LiPyphilic's AssignLeaflets tool,[S9] based on the positions of selected reference beads, GL1 and GL2 for phospholipids, and ROH for cholesterol, relative to the bilayer midplane. The midplane was determined by averaging the z coordinates of these beads.

The area per lipid was calculated using 2D Voronoi tessellation based on the x and y coordinates of the lipid headgroup beads: PO_4 for phospholipids and ROH for cholesterol. Calculations were performed using the `freud` software.[S10]

The calculation of the single coarse-grained bead chain order parameter was performed using the equation:[S11]

$$S_{chain} = \frac{\langle 3\cos^2\theta - 1\rangle}{2} \tag{1}$$

where θ was defined as the angle between the bilayer normal (z-axis) and the bond vector connecting two specific coarse-grained (CG) beads, ROH and C1, within cholesterol molecules, as illustrated in Figure S3. For the phospholipids, the two acyl chains of each

lipid, corresponding to the *sn*-1 and *sn*-2 positions on the glycerol backbone, were identified via bead selection. The order parameter, S_{CC}, was computed separately for each chain and then averaged for each lipid. For each simulation, the S_{CC} values were extracted at selected time frames during both heating (up) and cooling (down) phases. These values were averaged across all lipids to yield a mean S_{CC} per frame, providing a measure of overall tail order over time. The analysis was repeated across multiple sets and replicates to ensure statistical robustness.

Lipid translational order was analyzed using radial distribution functions (RDFs). In this analysis, the phospholipid tail beads C2A and C2B were selected as reference groups to characterize the structural organization of the lipid tails. RDFs were computed using the Gromacs gmx rdf tool. To focus on lateral organization within the membrane, the calculations were restricted to the in-plane RDFs, in the xy-plane.

Temperature, density profile and thermal transport calculations

To calculate the temperature and density profiles, the simulation boxes were divided into 200 equal slices along the z direction, with a width of δz. The temperature within each slice s is given by:[S12]

$$T(z) = \frac{1}{N_{dof,z} \cdot k_B} \sum_{i \in z} m_i v_i^2 \tag{2}$$

where the sum runs over all the beads in slice z, with m_i denoting the mass of bead i and v_i representing its speed. The total number of degrees of freedom for all the beads within a given slice is represented by $N_{dof,z}$, and k_B is the Boltzmann constant.

The number density (per nm^3) of beads was calculated using the equation,

$$\rho(z, A) = \frac{1}{A} \left\langle \sum_{i=1}^{N} \delta(z - z_i) \right\rangle \tag{3}$$

where A is the bilayer area and the sum runs over N beads. The angular brackets denote a

time average.

To calculate the temperature gradients (∇T), the simulation boxes were divided into sections to isolate the thermal gradient associated with the bilayer. The bilayer regions were defined between the maxima of the PO_4 density peaks of lipids in the two leaflets. The bilayer thickness was calculated as the average of the two distances. Temperature gradients within the bilayer were determined by fitting the combined temperature profiles of the hydrocarbon chains and cholesterol molecules to a linear function. The thermal conductivity (λ) of the bilayers was calculated using Fourier's law (see main paper). The thermal conductance of the bilayer was then obtained from the thermal conductivity and bilayer thickness (see main paper for a discussion).

Cholesterol partitioning

Figure S3 illustrates the definition of the order parameter θ, used to calculate the free energy surfaces presented in both the main article and this Supporting Information (see below).

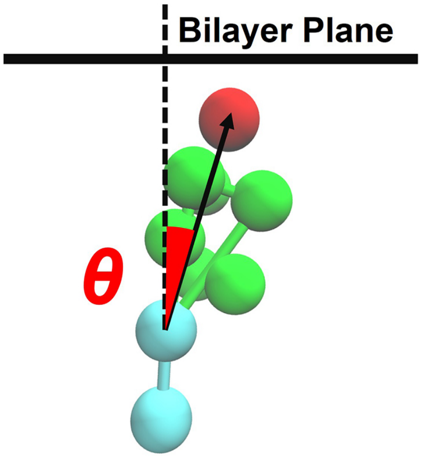

Figure S3: Definition of the tilt angle (θ) of cholesterol relative to the bilayer plane. The angle is defined with respect to the vector normal to the bilayer plane.

Under nonequilibrium conditions, cholesterol molecules were assigned to either the hot or cold leaflets based on the z coordinate of their ROH bead relative to the bilayer midplane. The fraction of cholesterol in each leaflet was defined as the number of CHOL molecules

at time t in the leaflet divided by the total number of cholesterol molecules present in the bilayer. See the main paper.

Energy conservation tests

The `nstlist` parameter in Gromacs determines how frequently the list of particle pairs within the cutoff distance for nonbonded interactions is updated. A large `nstlist` value can result in incorrect force calculations and discontinuous jumps in forces under nonequilibrium conditions, leading to total energy drift and loss of energy conservation. We performed test calculations using a DPPC:CHOL 50:50 lipid bilayer, with the thermostats set to 300 and 400 K. A time step of 0.02 ps was used, and the system consisted of 39,148 beads. To monitor the energy conservation, we calculated the percentage difference between the absolute values of the cumulative friction parameter of the Nosé-Hoover thermostat, which provides the cumulative energy exchanged at the thermostats (see reference[S13] for a discussion of the method using the Nosé-Hoover thermostat). The error increased with `nstlist`: 0.26, 5 and 97% for values of `nstlist = 1, 5, 20`, respectively. `nstlist=1` provided excellent energy conservation (see fig. S3), and it was used in all our NEMD simulations.

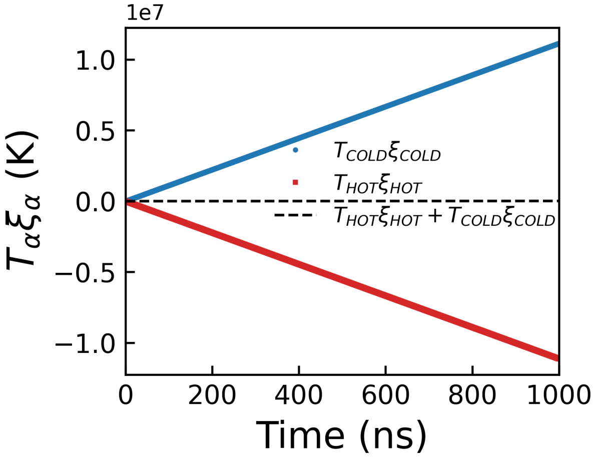

Figure S4: Cumulative heat exchanged at the hot (blue) and cold (red) thermostats, calculated as the cumulative friction parameter of the Nosé–Hoover thermostat and the thermostat temperature, plotted versus time for a DPPC:CHOL 50:50 bilayer. The results were obtained with `nstlist = 1`, resulting in excellent energy conservation, as shown by the dashed line, which represents the sum of the friction parameters. The sum is essentially zero.

The parameter *nstcomm* dictates the frequency with which the motion of the center of mass (CoM) of selected groups is removed. The effects of shifting CoMs became more evident as simulation runtimes increased. We tested four combinations of `nstlist` and `nstcomm` values using a 50:50 DPPC:CHOL mixture.

Initial tests on the 1 μs NEMD simulations using GROMACS version 2024.1 showed that `nstlist=1` combined with `nstcomm=10` resulted in a shift in the bilayer position along the z direction. Smaller shifts were observed with the parameters `nstlist=5`, `nstcomm=1` using GROMACS version 2024.1 and `nstlist = 1`, `nstcomm = 1` using GROMACS version 2021.3. The shift was significantly reduced (below 0.2 nm) in GROMACS 2024.1 when using `nstlist=1` and `nstcomm=1`. This setup was employed in our simulations.

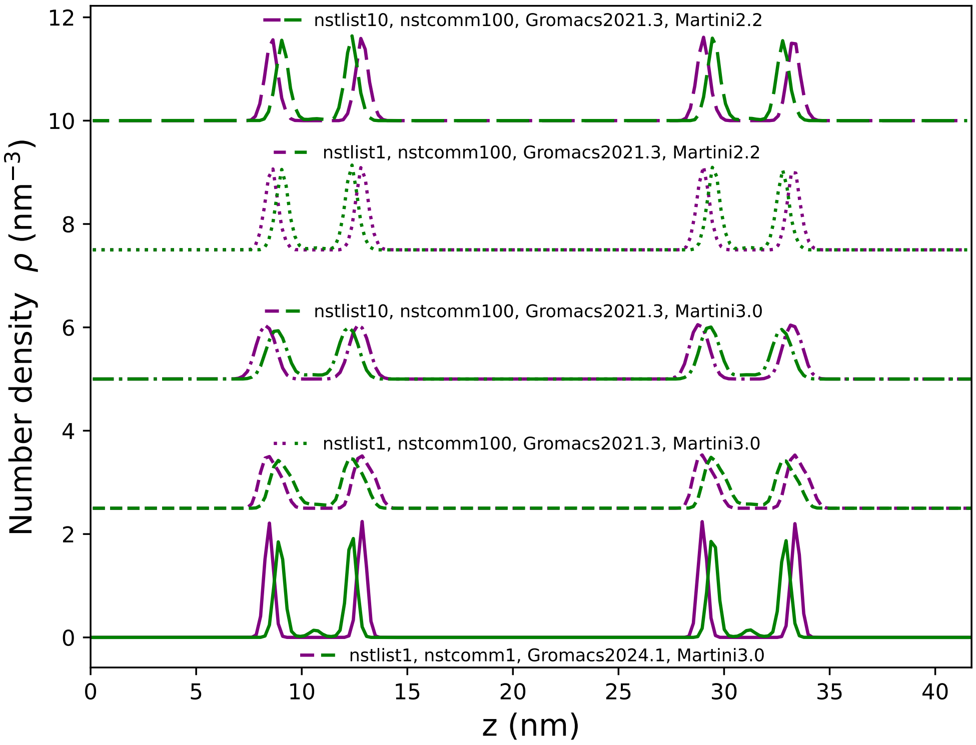

Figure S5: Number density of the PO_4 and ROH groups for a 50:50 DPPC:CHOL bilayer, obtained using different simulation parameters, GROMACS versions, and MARTINI force fields.

Phase transitions

To quantify the $S_{\mathrm{o}} \rightarrow L_{\mathrm{d}}$ transition, we plotted the average DPPC area (a_{DPPC}) and the average chain order parameter (S_{chain}) as functions of temperature (Figure S6). For pure DPPC bilayers, a_{DPPC} was found to be 0.478 ± 0.001 nm^2 at 280 K and 0.634 ± 0.016 nm^2 at 340 K, in good agreement with previous reports.[S11,S14,S15] Significant hysteresis loops were observed at low cholesterol mole fractions, indicating a first-order transition. The magnitude of the discontinuous jump decreases with increasing cholesterol mole fraction and disappears completely at mole fractions > 40mol%. At higher cholesterol mole fractions, a_{DPPC} remains nearly constant with temperature.

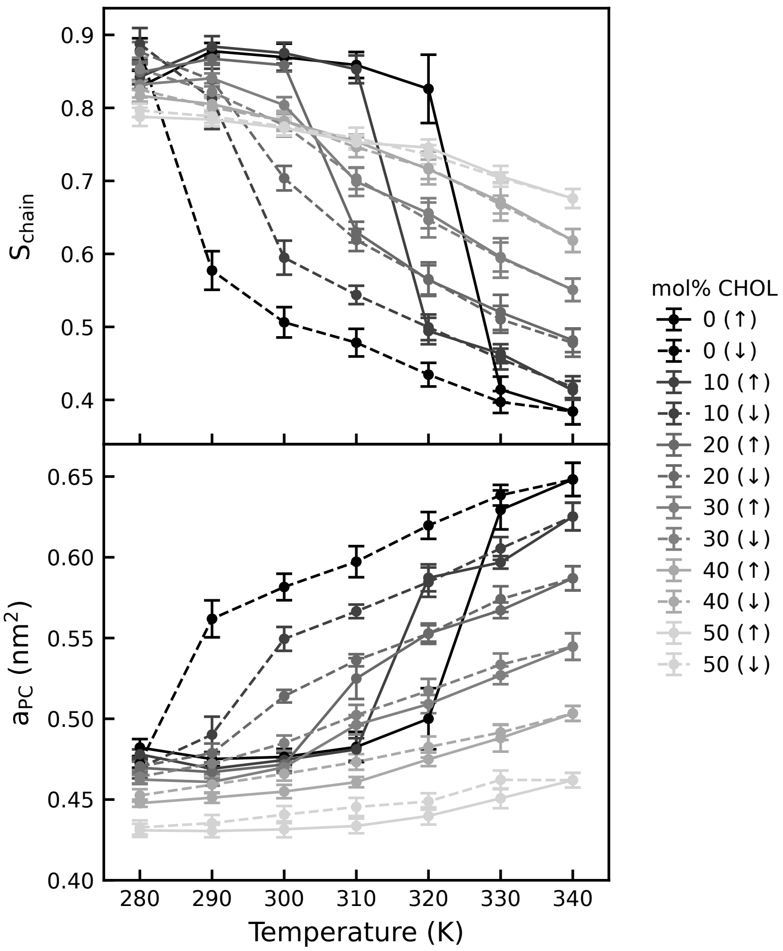

Figure S6: Average chain order parameter (top) and area per DPPC lipid (bottom) calculated using the Voronoi tessellation method, from the heating (solid lines) and cooling (dashed lines) cycles, plotted as a function of temperature for different mole fractions of cholesterol. Upward and downward arrows in the legend indicate the heating and cooling cycles, respectively. Each error bar represents the standard deviation of the mean computed across ten independent replicates, using 50 ns equilibrium trajectories as described on page S-6. The temperatures on the x-axis correspond to the target temperatures of these equilibrium simulations.

The melting temperature T_m was estimated using the hysteresis method:[S16–S18]

$$T_m = T_{\text{liquid}\to\text{gel}} + T_{\text{gel}\to\text{liquid}} - \sqrt{T_{\text{liquid}\to\text{gel}} \times T_{\text{gel}\to\text{liquid}}} \tag{4}$$

where $T_{\text{gel}\to\text{liquid}}$ and $T_{\text{liquid}\to\text{gel}}$ are the phase transition temperatures during the heating and cooling cycles, respectively.

A transition temperature of approximately 306–308 K was observed for pure DPPC. This is close to those reported in previous MARTINI 2 and MARTINI 3 studies.[S11,S17,S19] Among the phospholipids investigated in this study, DPPC exhibited the highest melting temperature (T_m). The experimental values for pure DPPC range between 311 and 314 K.[S20,S21] In contrast, the T_m for POPC is 268–271 K,[S22,S23] and for DLiPC, 216 K.[S24] The lower T_m values observed for unsaturated lipids are attributed to the presence of double bonds, which introduce kinks in the hydrocarbon chains and disrupt tight packing.

Wang *et al.*[S19] used a volume-based method and found the a_{DPPC} increases with increasing cholesterol mole fraction at low temperatures, whereas Zhang *et al.*[S11] found the opposite at all temperatures using the Voronoi tessellation method. Our results agree with those of the latter.

Analysis of the 2D radial distribution functions (see Figure S7), confirms the absence of a first-order phase transition at cholesterol mole fractions > 40 mol%. Systems exhibiting higher translational order displayed extended oscillatory behavior in their RDF profiles, whereas those with elevated cholesterol mole fractions (≥ 40 mol%) showed minimal temperature dependence. The RDF of pure DPPC exhibits a significant change in long-range structure around 300 K, close to the estimated melting temperature (see above). The positions and heights of the first, second, and third RDF peaks are reported in Table S6 Increasing the temperature reduces peak heights. At 300 K and above, cholesterol increases the RDFs' peak heights, with only small shifts in peak positions.

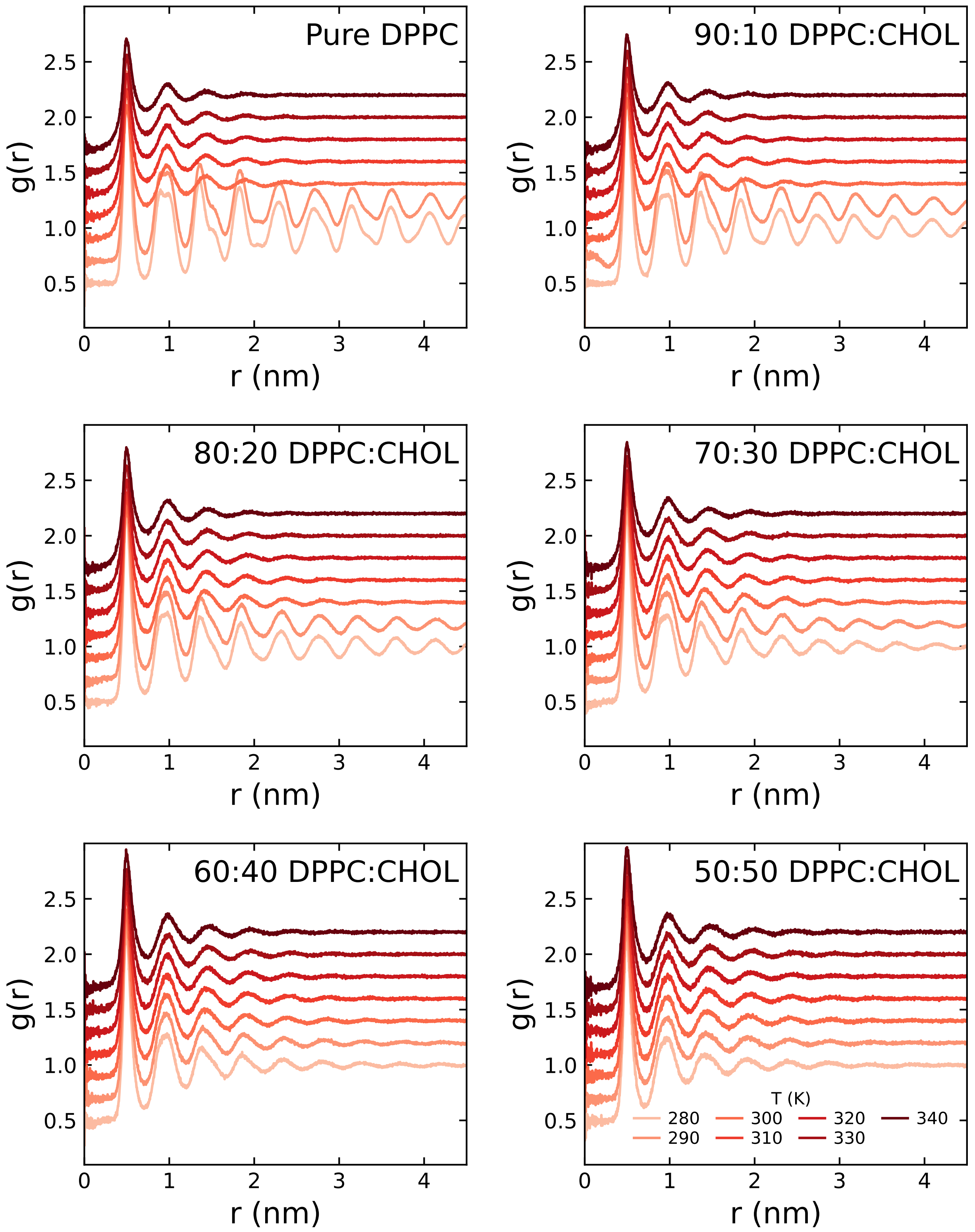

Figure S7: 2D-RDFs of DPPC C2A and C2B beads for each composition at different temperatures. Each dataset for T > 280 K has been shifted vertically by 0.2 units for clarity.

To probe the $S_o \rightarrow L_o$ and $L_d \rightarrow L_o$ transitions, we plotted S_{chain} versus mol% CHOL (Figure S8). No discontinuities were observed, supporting Zhang et al.'s[S11] conclusion that these transitions are weak and likely second order. The structural changes between 300 and 310 K in Figure S8 (bottom) support the estimated transition temperature.

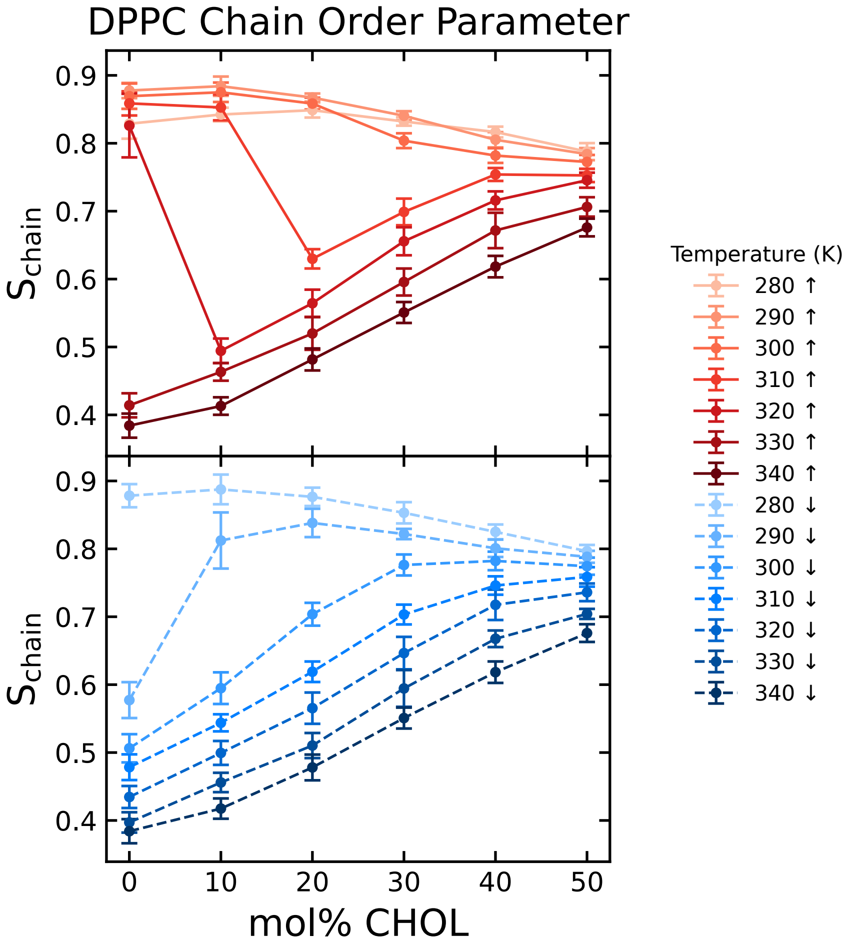

Figure S8: DPPC chain order parameter from the heating cycle (top) and cooling (dashed lines) cycles (bottom), plotted as a function of cholesterol mole fraction. Upward and downward arrows in the legend indicate the heating and cooling cycles, respectively. Each error bar represents the standard deviation of the mean computed across ten independent replicates, using 50 ns equilibrium trajectories as described on page S-5. The temperatures in the legend correspond to the target temperatures of these equilibrium simulations.

The average area per lipid was calculated across all temperatures and compositions using 2D Voronoi tessellation method. As shown in Figure S9, the average area per lipid decreases systematically with increasing cholesterol mole fraction at all temperatures. This trend

arises from two complementary factors: the condensing effect of cholesterol and cholesterol's smaller molecular footprint compared to phosphatidylcholine lipids.

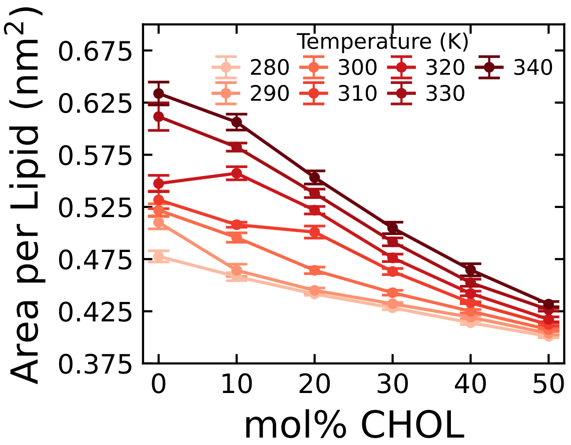

Figure S9: Average area per lipid calculated using the 2D Voronoi tessellation method for a DPPC bilayer as a function of cholesterol content. Each error bar represents the standard deviation of the mean computed across ten independent replicates, using 50 ns equilibrium trajectories as described on page S-5. The temperatures on the legend correspond to the target temperatures of these equilibrium simulations.

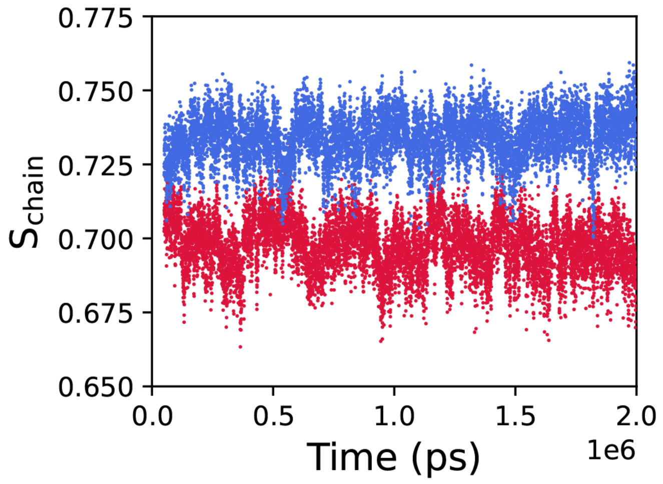

Figure S10: Time evolution of DPPC chain order parameters in the cold leaflet (blue) at $\sim$ 337 K and hot leaflet (red) at $\sim$ 357 K for the 50:50 DPPC:CHOL system in the NEMD production simulations.

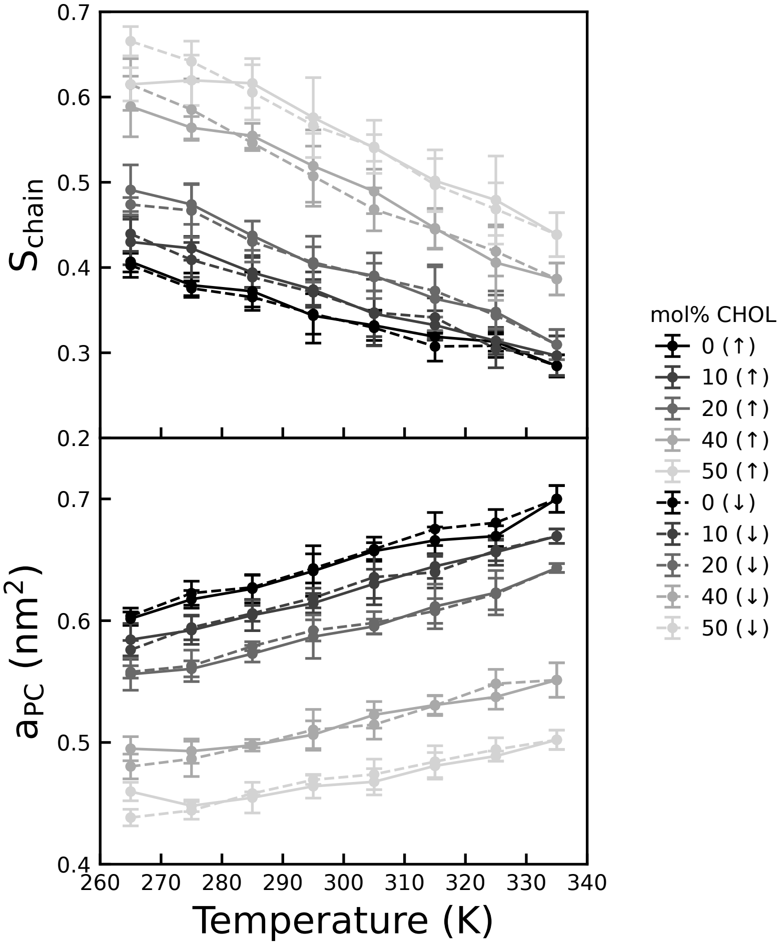

Figure S11: Average chain order parameter (top) and area per POPC lipid (bottom), obtained using the Voronoi tessellation method from heating (solid lines) and cooling (dashed lines) cycles, plotted as a function of temperature for different cholesterol mol%. Each error bar represents the standard deviation calculated from the mean of the ten replicates.

Temperature and Density Profiles

Temperature and density profiles were analyzed for bilayer systems containing 10 and 50 mol% cholesterol (Figure S12 and Figure S13). Spatial temperature distributions were resolved for water, phosphatidylcholine (PC) hydrocarbon chains, and cholesterol. Initial analyses using 200 spatial slices revealed substantial fluctuations in the temperature profiles. To reduce fluctuations, we employed coarse binning (50 bins) with a 4-point running average.

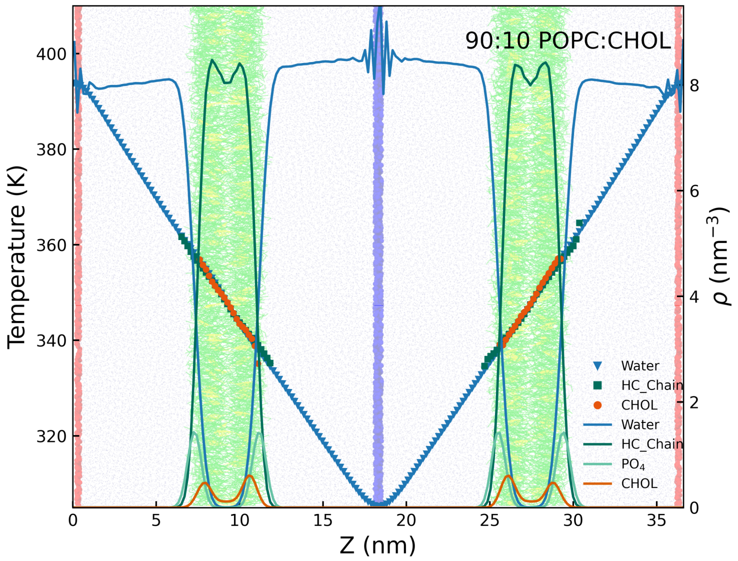

Figure S12: Temperature (markers and left vertical axis) and bead density (in beads per nm^{-3} profiles (lines and right vertical axis) for selected groups in POPC bilayers containing 10 mol% cholesterol, averaged over five replicas. The data are overlaid over a system snapshot. "HC-Chain" in the legend denotes the hydrocarbon chains of the PC lipid, "CHOL" the cholesterol molecules and "Water" the water beads. Water beads in the hot and cold thermostats are highlighted in red and blue colors, respectively.

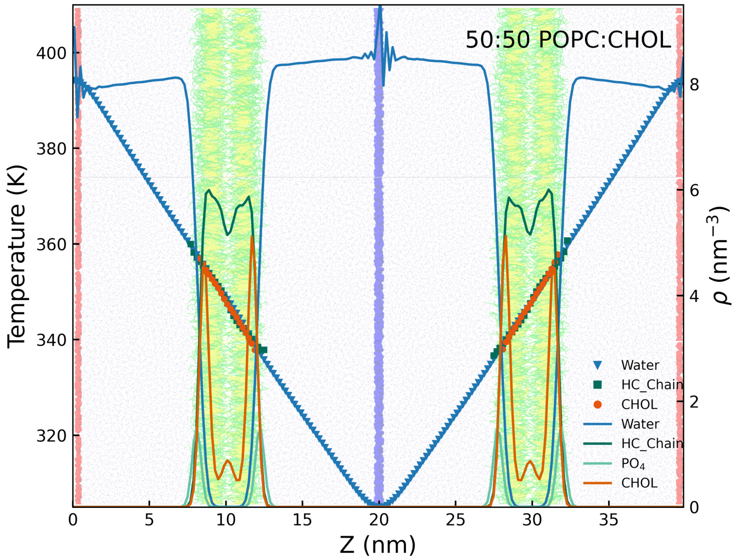

Figure S13: Temperature and density profiles for POPC bilayers with 50 mol% cholesterol, averaged over five replicas. Symbols are defined in Figure 12. The data are overlaid over a system snapshot.

Two pronounced density maxima emerge at the DBPC:CHOL and DXPC:CHOL bilayer

midplane, corresponding to the interdigitation of hydrocarbon chain termini from opposing leaflets.

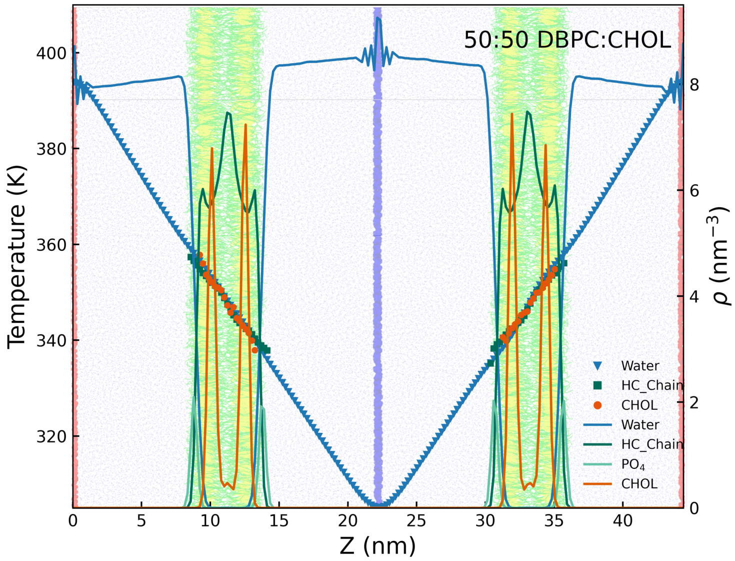

Figure S14: Temperature and density profiles for DBPC bilayers with 50 mol% cholesterol, averaged over five replicas. Symbols are defined in Figure 12. The temperature and density profiles are overlaid on a system snapshot.

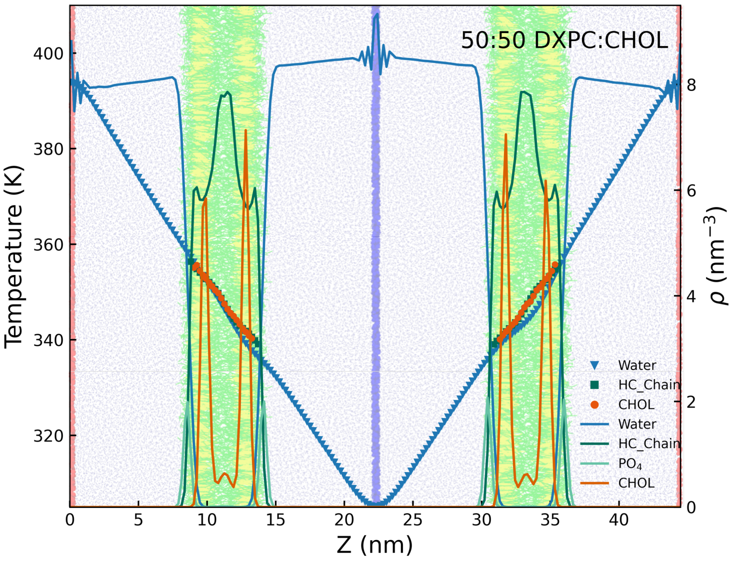

Figure S15: Temperature and density profiles for DXPC bilayers with 50 mol% cholesterol, averaged over five replicas. Symbols are defined in Figure S12.The temperature and density profiles are overlaid on a system snapshot.

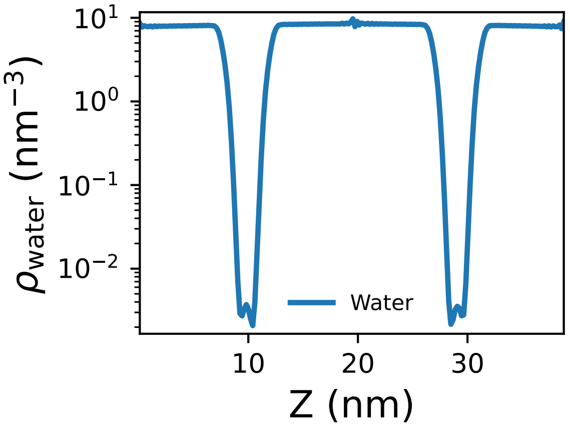

Figure S16: Water density profiles in DPPC bilayers with 10 mol% cholesterol, shown on a logarithmic scale and averaged over five independent replicas.

Center of mass (COM) displacement

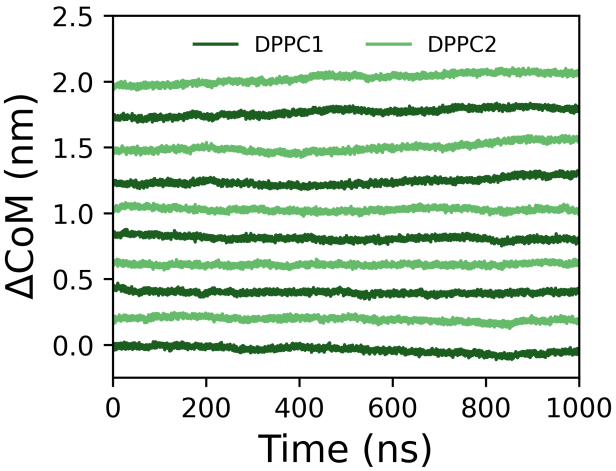

Figure S17: Time evolution of the center-of-mass displacement along the z direction relative to the initial configuration for DPPC in DPPC:CHOL (50:50) bilayers during the NEMD production stage. Individual replica trajectories are vertically offset by a constant increment of $0.03\,\mathrm{nm\,ns^{-1}}$ from replica 1 to 5 (bottom to top) for visual clarity. Index 1 denotes molecules located in the bilayer near the box origin, whereas index 2 denotes molecules in the opposing bilayer near the upper boundary of the simulation box along z.

As shown in Figure S17, with the whole-system centre-of-mass motion being removed at each step, the CoM along z under the imposed temperature does not show systematic drift.

Bilayer thickness

The ordering effect of cholesterol on hydrocarbon chains is evident from bilayer thicknesses (Figure S18), defined as the distance between the maxima in the PO_4 density profiles of the two bilayer leaflets. The thickness generally increases with cholesterol mole fraction, consistent with previous reports.[S19,S25,S26]

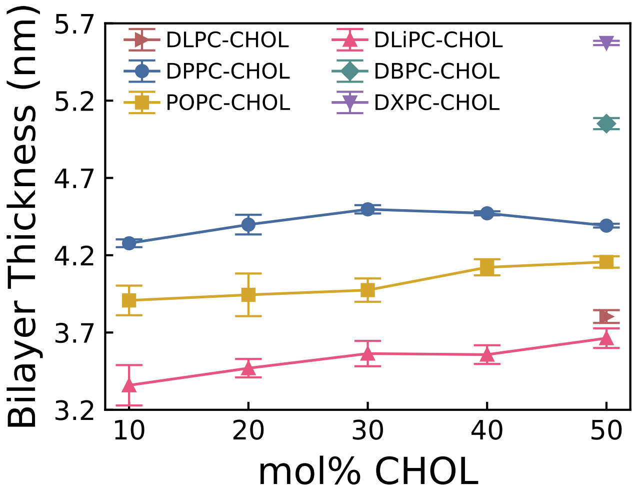

Figure S18: Bilayer thickness as a function of cholesterol mole fraction for various phospholipid bilayers. For DLPC, DBPC, and DXPC, data are shown for a single cholesterol mole fraction of 50 mol%. Error bars denote the standard deviation across the five sampled trajectories.

Table S3: Bilayer parameters calculated from temperature and density profiles with standard deviation, averaged over five replicas per system. [a]Bilayer thickness. [b]Temperature difference between the hot and cold leaflets, calculated from the temperature of the PO_4 headgroups, and [c]Thermal gradient.

**Membrane Composition**	$\delta z_{\text{bilayer}}$ (nm)[a]	$\Delta T_{\text{bilayer}}$ (K)[b]	$\nabla T_{\text{bilayer}} \times 10^9$ (K m^{-1})[c]
90:10 DPPC:CHOL	4.277 ± 0.025	18 ± 0.17	4.528 ± 0.139
80:20 DPPC:CHOL	4.398 ± 0.064	16 ± 0.25	4.112 ± 0.136
70:30 DPPC:CHOL	4.497 ± 0.027	16 ± 0.26	3.959 ± 0.160
60:40 DPPC:CHOL	4.471 ± 0.013	16 ± 0.19	4.087 ± 0.161
50:50 DPPC:CHOL	4.392 ± 0.012	18 ± 0.25	4.357 ± 0.126
90:10 POPC:CHOL	3.908 ± 0.096	18 ± 0.15	5.254 ± 0.191
80:20 POPC:CHOL	3.944 ± 0.138	18 ± 0.00	5.058 ± 0.087
70:30 POPC:CHOL	3.974 ± 0.076	18 ± 0.19	5.071 ± 0.119
60:40 POPC:CHOL	4.122 ± 0.052	19 ± 0.11	5.016 ± 0.144
50:50 POPC:CHOL	4.156 ± 0.037	19 ± 0.04	5.148 ± 0.092
90:10 DLiPC:CHOL	3.359 ± 0.131	19 ± 0.05	6.055 ± 0.275
80:20 DLiPC:CHOL	3.469 ± 0.060	20 ± 0.36	6.206 ± 0.257
70:30 DLiPC:CHOL	3.564 ± 0.082	20 ± 0.36	6.221 ± 0.174
60:40 DLiPC:CHOL	3.557 ± 0.061	21 ± 0.19	6.600 ± 0.140
50:50 DLiPC:CHOL	3.663 ± 0.064	21 ± 0.71	6.577 ± 0.083
50:50 DLPC:CHOL	3.783 ± 0.036	17 ± 0.74	5.092 ± 0.241
50:50 DLPC:CHOL	3.803 ± 0.042	22 ± 0.60	6.416 ± 0.189
50:50 DBPC:CHOL	5.051 ± 0.033	18 ± 0.03	3.996 ± 0.070
50:50 DBPC:CHOL	5.051 ± 0.036	21 ± 0.23	4.936 ± 0.030
50:50 DXPC:CHOL	5.573 ± 0.014	18 ± 0.07	3.772 ± 0.087
50:50 DXPC:CHOL	5.573 ± 0.014	24 ± 0.26	4.734 ± 0.065

Heat flux, thermal conductivity and thermal conductance

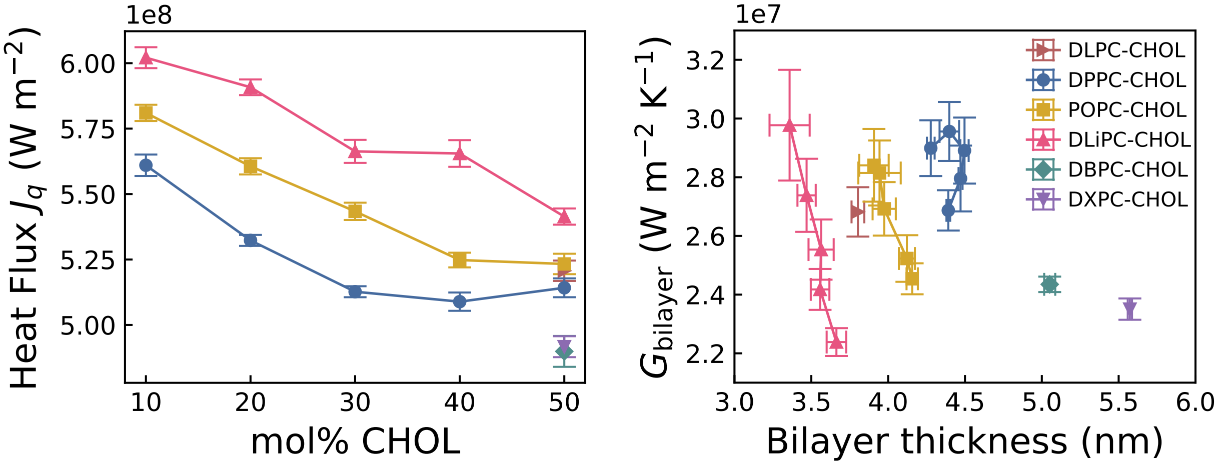

(a) (b)

Figure S19: (a) Heat flux as a function of cholesterol mole fraction (mol %) and (b) thermal conductance as a function of bilayer thickness for various phospholipid bilayers. For DLPC, DBPC, and DXPC systems, data are shown for a single cholesterol mole fraction of 50 mol%. Error bars denote the standard deviation across the five sampled trajectories. The legend defining the symbols used in panels (a) and (b) is shown in panel (b).

Table S4: Bilayer thermal properties averaged over five replicas per system. All the heat flux values, J_q, have a percentage standard deviation $< 1\%$.

**Membrane Composition**	$J_q \times 10^8$ (W m^{-2})	$\lambda_{bilayer}$ (W m^{-1} K^{-1})	$G_{bilayer} \times 10^7$ (W m^{-2} K^{-1})
90:10 DPPC:CHOL	5.610	0.124 ± 0.004	2.899 ± 0.095
80:20 DPPC:CHOL	5.323	0.130 ± 0.004	2.956 ± 0.100
70:30 DPPC:CHOL	5.127	0.130 ± 0.005	2.891 ± 0.113
60:40 DPPC:CHOL	5.089	0.125 ± 0.005	2.796 ± 0.112
50:50 DPPC:CHOL	5.142	0.118 ± 0.003	2.687 ± 0.069
90:10 POPC:CHOL	5.810	0.111 ± 0.004	2.841 ± 0.124
80:20 POPC:CHOL	5.606	0.111 ± 0.002	2.814 ± 0.111
70:30 POPC:CHOL	5.434	0.107 ± 0.003	2.692 ± 0.091
60:40 POPC:CHOL	5.248	0.104 ± 0.003	2.523 ± 0.079
50:50 POPC:CHOL	5.233	0.102 ± 0.002	2.454 ± 0.053
90:10 DLiPC:CHOL	6.021	0.100 ± 0.005	2.977 ± 0.189
80:20 DLiPC:CHOL	5.908	0.095 ± 0.004	2.738 ± 0.124
70:30 DLiPC:CHOL	5.663	0.091 ± 0.003	2.553 ± 0.103
60:40 DLiPC:CHOL	5.655	0.086 ± 0.002	2.418 ± 0.070
50:50 DLiPC:CHOL	5.414	0.082 ± 0.001	2.238 ± 0.047
50:50 DLPC:CHOL	5.207	0.103 ± 0.005	2.723 ± 0.135
50:50 DLPC:CHOL	6.528	0.102 ± 0.003	2.682 ± 0.084
50:50 DBPC:CHOL	4.899	0.123 ± 0.002	2.435 ± 0.043
50:50 DBPC:CHOL	6.140	0.123 ± 0.001	2.435 ± 0.026
50:50 DXPC:CHOL	4.917	0.130 ± 0.003	2.333 ± 0.054
50:50 DXPC:CHOL	6.180	0.131 ± 0.002	2.351 ± 0.036

Cholesterol flip-flop

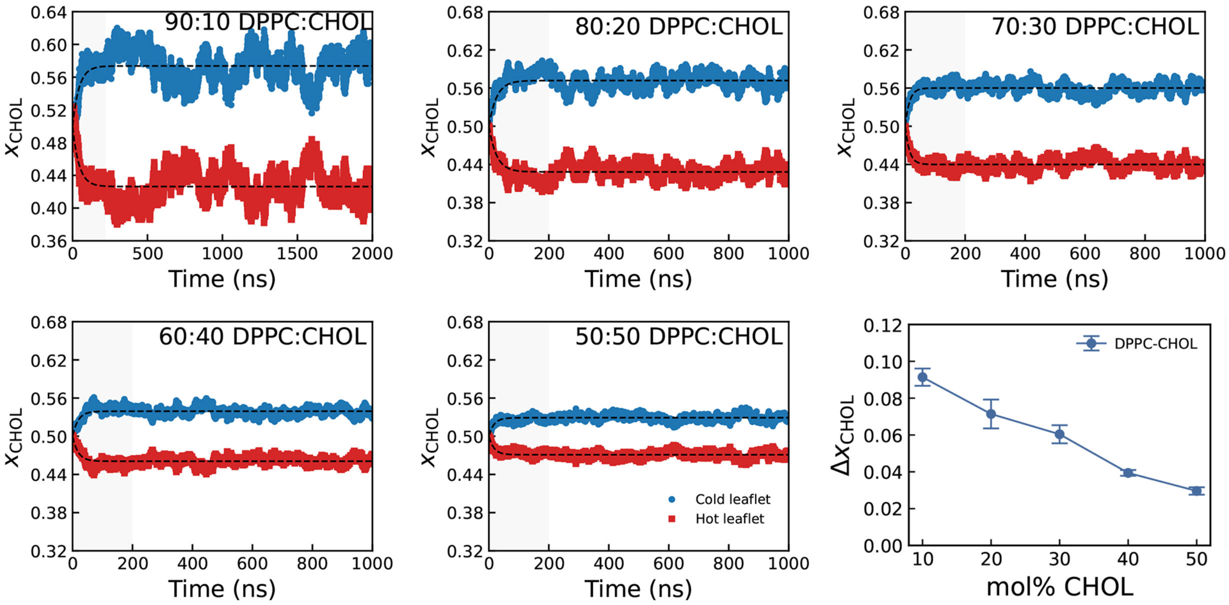

Figure S20: Time-dependent fraction of cholesterol in the hot (red) and cold (blue) leaflets for DPPC bilayers with different cholesterol contents. Data for each system were averaged over five independent replicas. The bottom-right panel shows the difference between the cholesterol fraction in the cold leaflet at steady state and the initial fraction at the beginning of the simulation (0.5). Error bars denote the standard deviation across the five sampled trajectories.

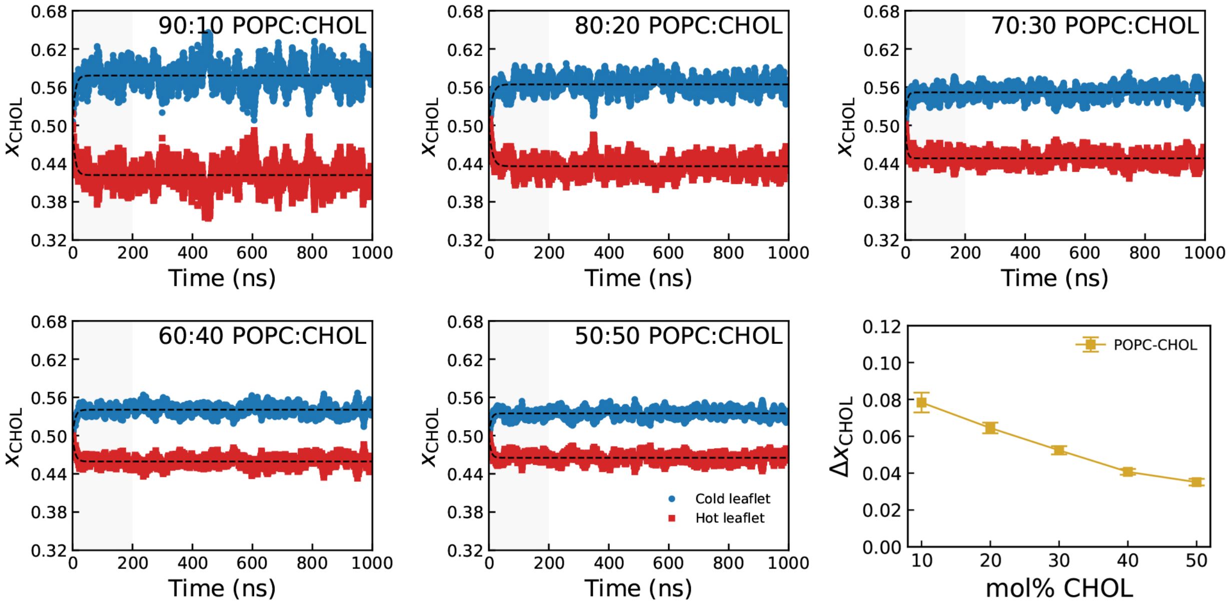

Figure S21: Same as Figure S20 for POPC bilayers.

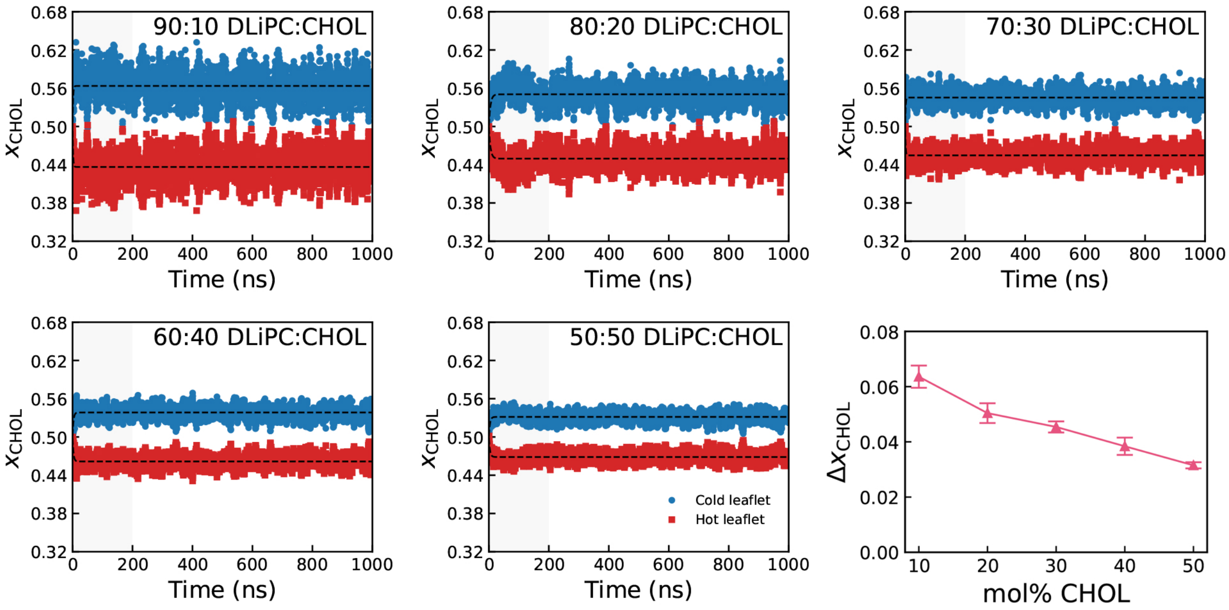

Figure S22: Same as Figure S20 for DLiPC bilayers.

Figure S23 shows the change in the fraction of cholesterol molecules, Δx_{CHOL} of the cold leaflet relative to the initial value of cholesterol in that leaflet 0.5. The results correspond to the 50:50 PC:CHOL composition. The data indicate an increase in cholesterol enrichment with increasing temperature gradient, consistent with a linear response in cholesterol transport across the bilayer.

In the linear-response regime, S_T can be estimated from these data using the steady-state relation in the main text together with the system-specific membrane temperature drop ΔT. For $\Delta T \approx 9$ K as a representative example, we obtain $S_T = 4.6 \times 10^{-3}$ K^{-1} for DXPC and 3.1×10^{-3} K $^{-1}$ for DPPC. These values are in good agreement with the data reported in Table S5 (Figure 9 in the main paper), indicating only a weak temperature dependence of the Soret coefficient over the explored temperature gradients and consistency with a linear response description.

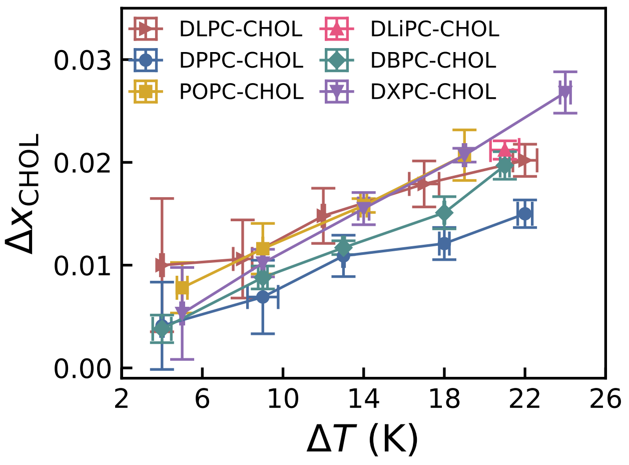

Figure S23: Change in the steady-state cholesterol fraction in the cold leaflet from the initial configuration is plotted against the local temperature difference across the bilayer. Error bars denote the standard deviation across the five sampled trajectories. All the systems correspond to a composition of 50:50 PC:CHOL.

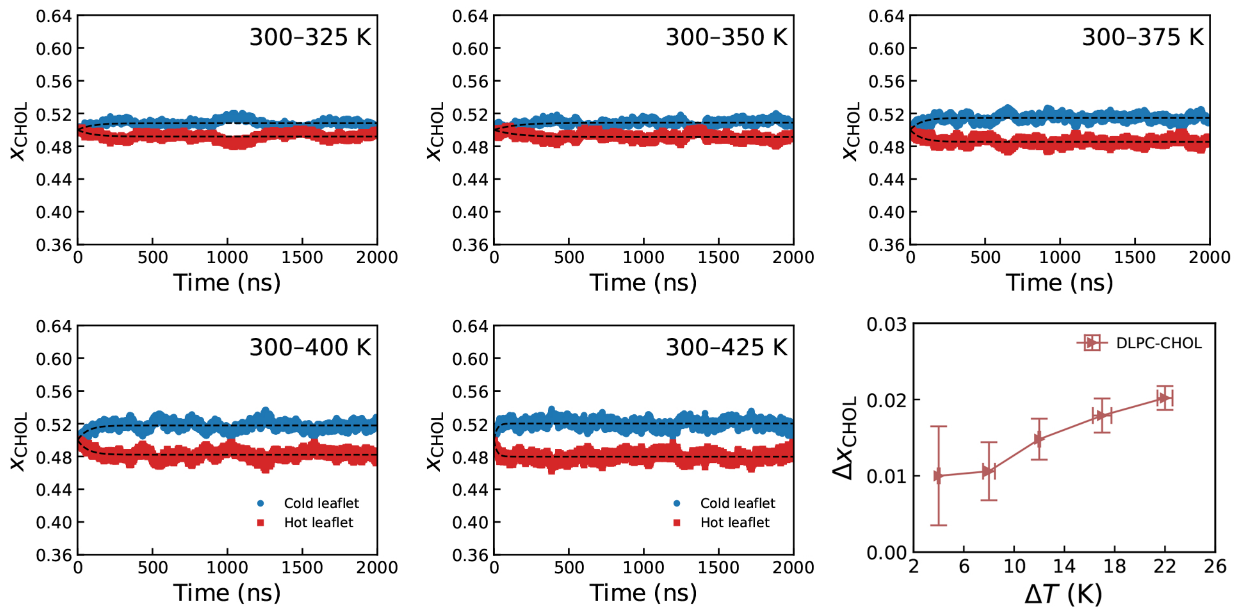

Figure S24: Same as Figure S20, but for DLPC bilayers under different temperature gradients. All data correspond to the 50:50 PC:CHOL composition.

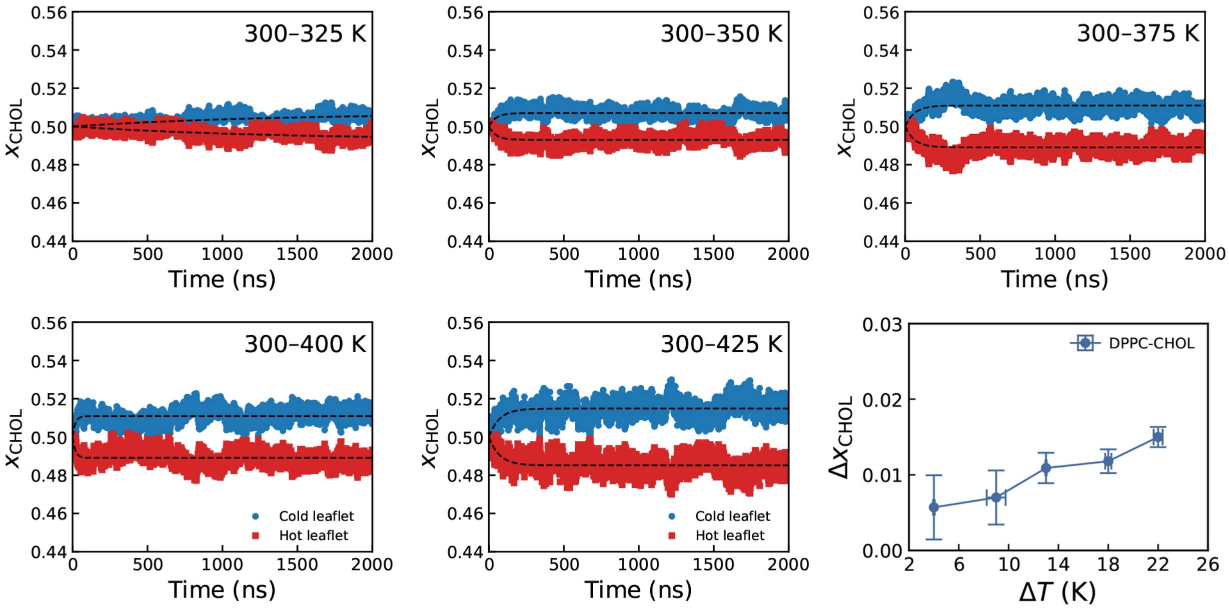

Figure S25: Same as Figure S24 for DPPC bilayers.

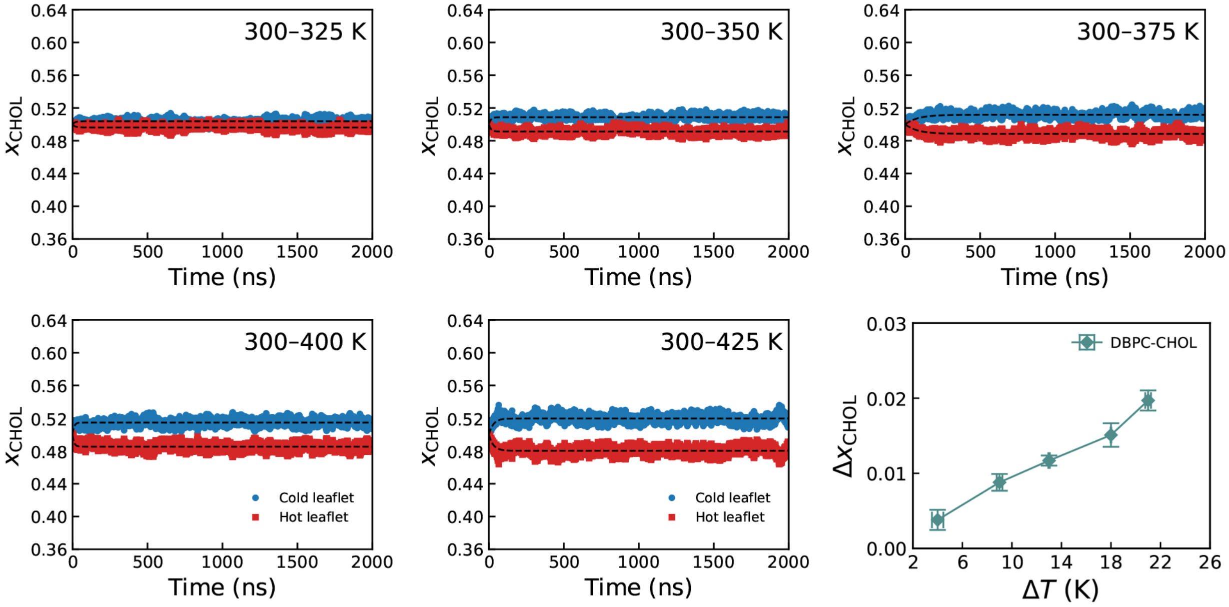

Figure S26: Same as Figure S24 for DBPC bilayers.

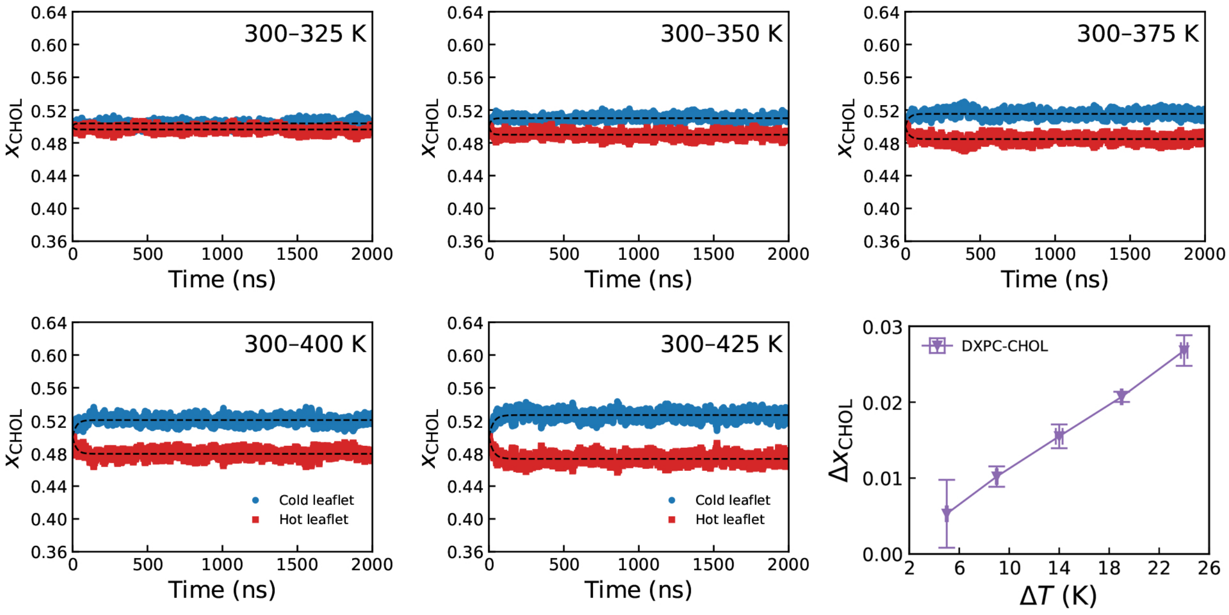

Figure S27: Same as Figure S24 for DXPC:CHOL 50:50 bilayers.

Soret coefficients

Table S5: Cholesterol flip-flop parameters averaged over five replicas per system.

Composition	$\Delta T_{bilayer}$ (K)	S_T (10^{-2} K^{-1})
90:10 DPPC:CHOL	18 ± 0.17	1.669 ± 0.299
80:20 DPPC:CHOL	16 ± 0.25	1.180 ± 0.130
70:30 DPPC:CHOL	16 ± 0.26	0.703 ± 0.129
60:40 DPPC:CHOL	16 ± 0.19	0.395 ± 0.062
50:50 DPPC:CHOL	18 ± 0.25	0.262 ± 0.030
90:10 POPC:CHOL	18 ± 0.15	1.271 ± 0.115
80:20 POPC:CHOL	18 ± 0.00	0.951 ± 0.080
70:30 POPC:CHOL	18 ± 0.19	0.700 ± 0.036
60:40 POPC:CHOL	19 ± 0.11	0.518 ± 0.024
50:50 POPC:CHOL	19 ± 0.04	0.438 ± 0.038
90:10 DLiPC:CHOL	19 ± 0.05	0.933 ± 0.057
80:20 DLiPC:CHOL	20 ± 0.36	0.682 ± 0.104
70:30 DLiPC:CHOL	20 ± 0.36	0.590 ± 0.033
60:40 DLiPC:CHOL	21 ± 0.19	0.503 ± 0.026
50:50 DLiPC:CHOL	21 ± 0.71	0.404 ± 0.022
50:50 DLPC:CHOL	17 ± 0.74	0.421 ± 0.056
50:50 DBPC:CHOL	18 ± 0.03	0.336 ± 0.035
50:50 DXPC:CHOL	19 ± 0.07	0.436 ± 0.014

Free Energy Surfaces

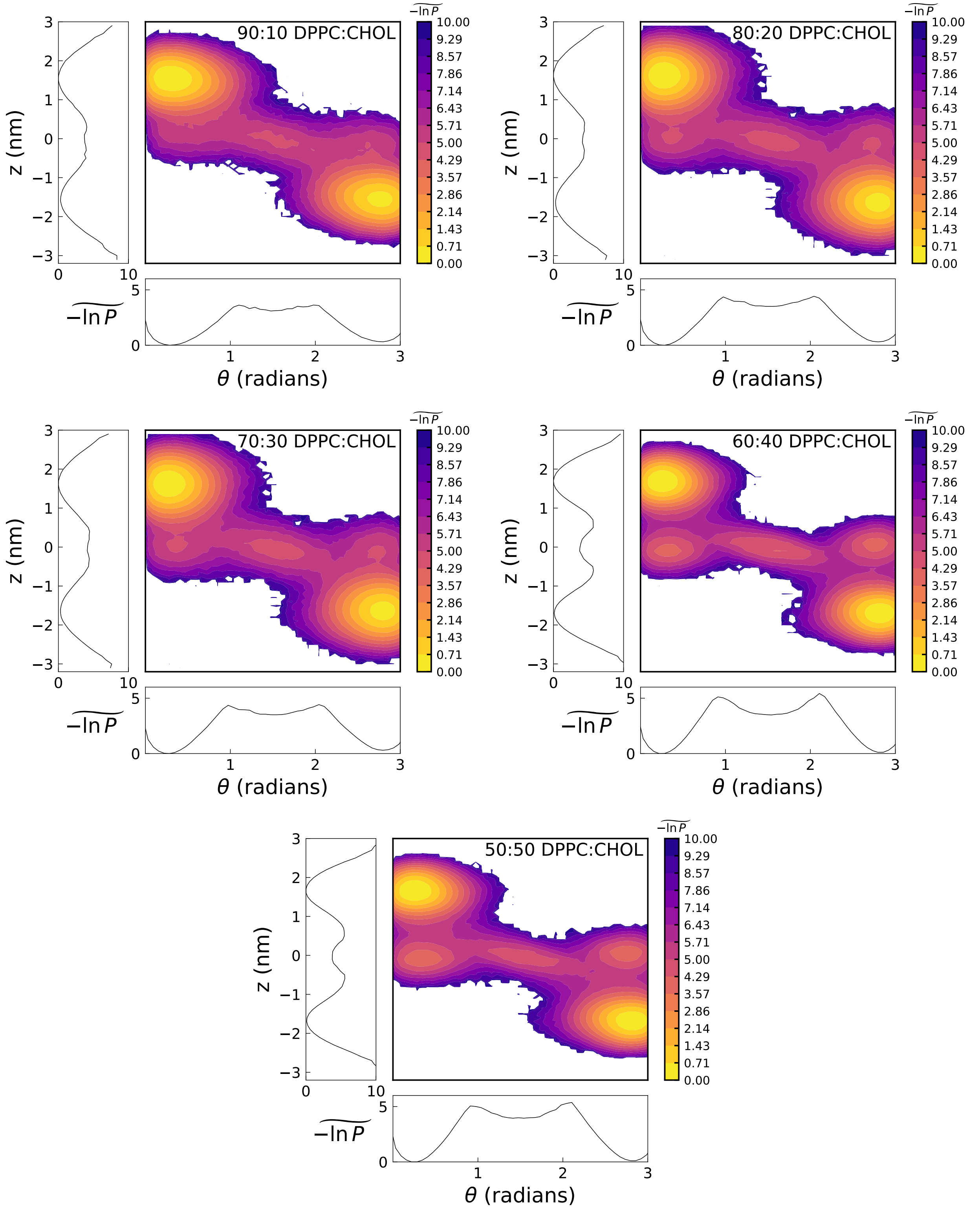

Figure S28: Free energy surface of DPPC bilayers containing 10 to 50 mol% mole fraction of cholesterol. The additional panels on the left and bottom of each plot represent $-\ln \widetilde{P(z,\theta)}$ projected along the z or θ order parameters. See main text for further details.

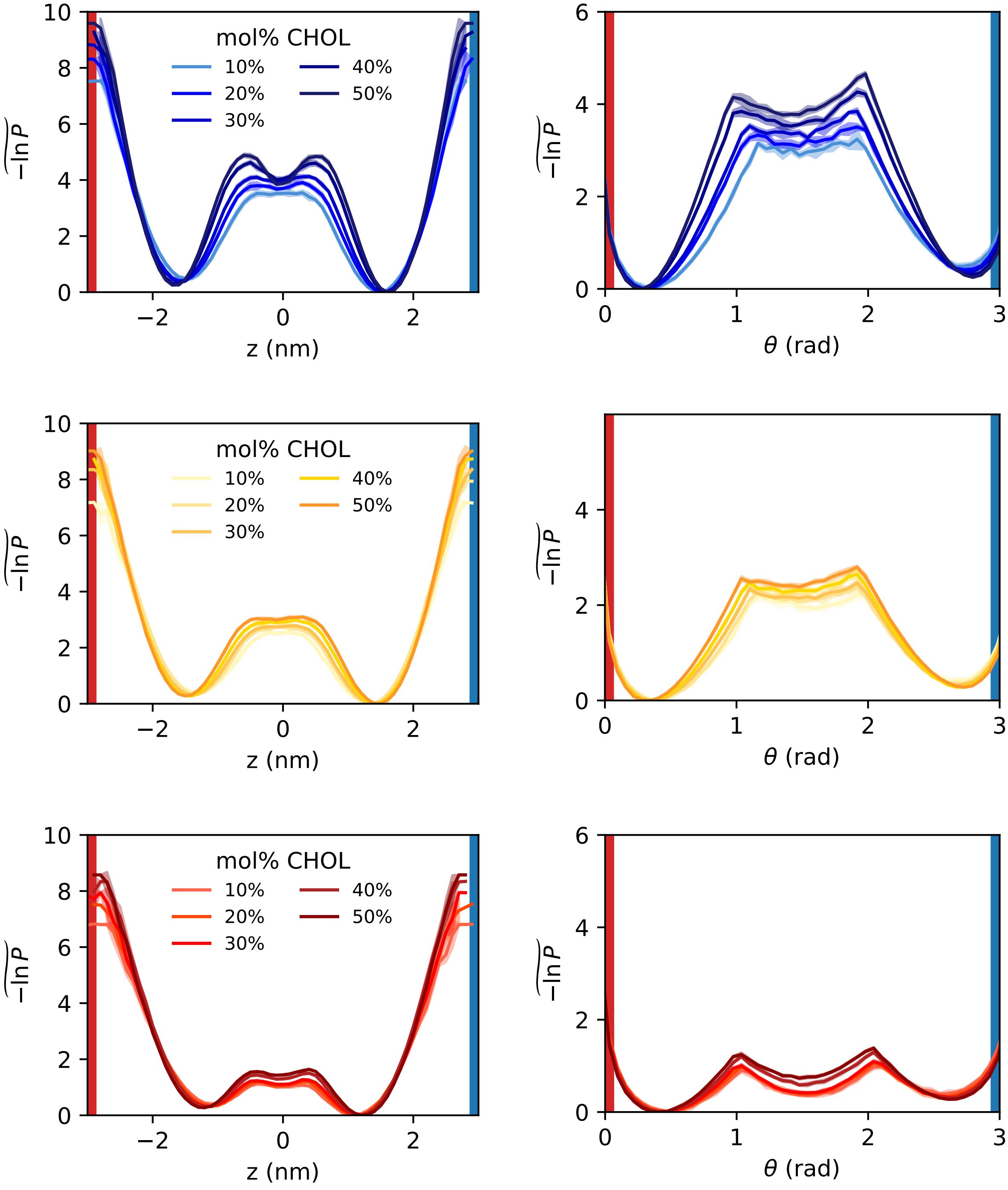

Figure S29: Free energy profiles represented as $-\ln \widetilde{P(z,\theta)}$ versus z (left panels) and θ (right panels) for DPPC (blue), POPC (yellow) and DLiPC (red) bilayers containing 10 to 50 mol% mole fraction of cholesterol. The blue and red vertical bars indicate the location of the cold and hot leaflets. Shaded regions indicate the statistical uncertainty, shown as the standard deviation.

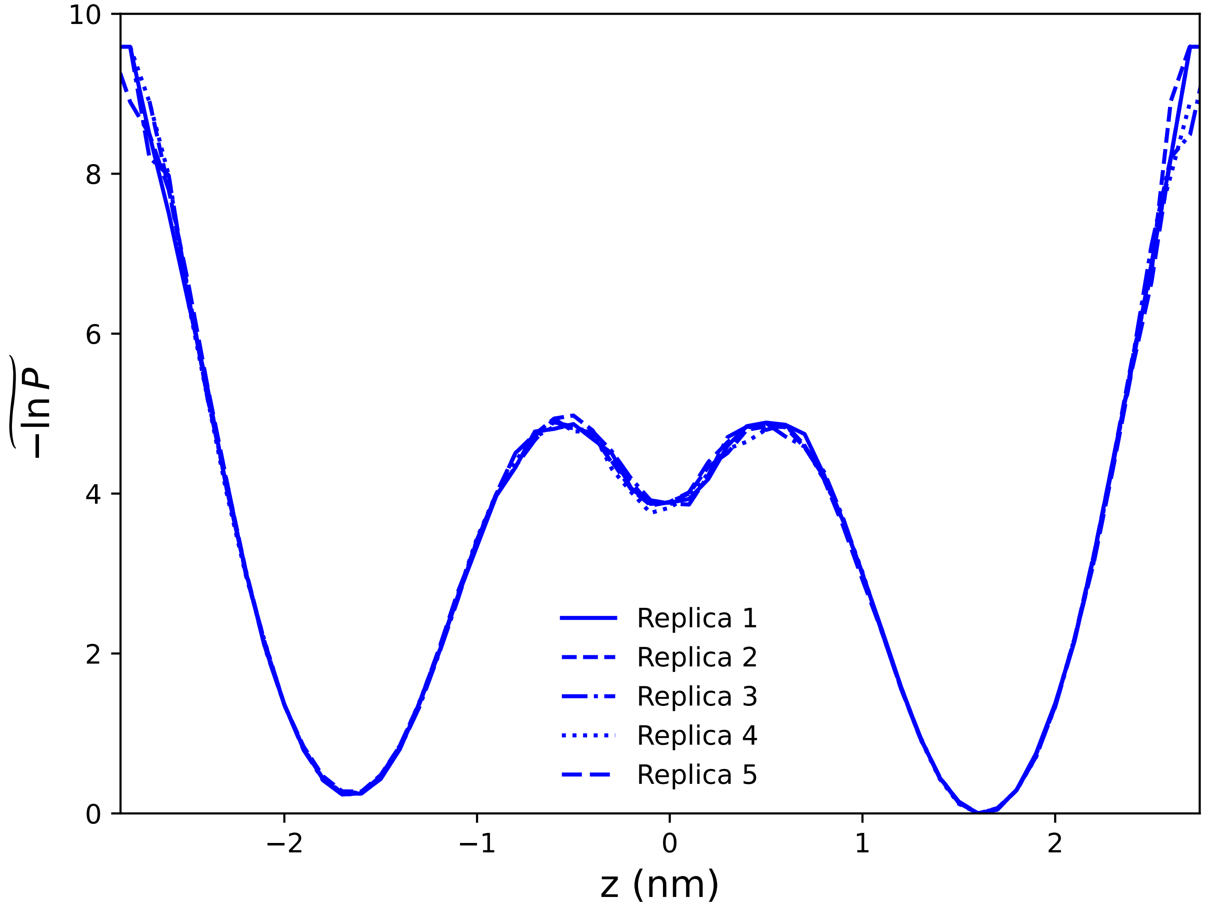

Figure S30: Free energy profiles represented as $-\ln \widetilde{P(z,\theta)}$ versus z (left panels) and θ (right panels) for the DPPC bilayer containing 50% cholesterol for each of the independent replica.

Kinetic model for analyzing cholesterol translocation

We assume that cholesterol translocation is a reversible process involving two rate constants. This assumption is consistent with the presence of two large activation barriers, while the movement of cholesterol from the metastable state in the middle of the bilayer involves, as shown in the main paper, a much smaller energy barrier.

$$\mathrm{N}_{\mathrm{CHOL,c}} \underset{k_{hc}}{\overset{k_{ch}}{\rightleftharpoons}} \mathrm{N}_{\mathrm{CHOL,h}} \tag{5}$$

where k_{ch} is the rate constant for the forward process, cold to hot ($N_{CHOL,c} \rightarrow N_{CHOL,h}$), and k_{hc} is the rate constant for the reverse process, hot to cold ($N_{CHOL,h} \rightarrow N_{CHOL,c}$). Here, $N_{CHOL,\alpha}$ denotes the number of cholesterol molecules in the cold ($\alpha = c$) or hot ($\alpha = h$) leaflet, such that $N_{CHOL,c} + N_{CHOL,h} = N_{CHOL,T}$, where $N_{CHOL,T}$ is the total number of cholesterol molecules in the bilayer.

The rate of change of $N_{CHOL,c}$ is given by:

$$\frac{dN_{CHOL,c}}{dt} = -k_{ch}N_{CHOL,c} + k_{hc}N_{CHOL_h} \tag{6}$$

and $dN_{CHOL,h}/dt = -dN_{CHOL,c}/dt$. Using conservation of the number of cholesterols we get,

$$\frac{dN_{CHOL,c}}{dt} = -(k_{ch} + k_{hc})N_{CHOL,c} + k_{hc}N_{CHOL,T} \tag{7}$$

In the stationary state, $dN^{s}_{CHOL,c}]/dt = 0$, and $N^{s}_{CHOL,c} = k_{hc}N_{CHOL,T}/(k_{ch} + k_{hc})$, where N^s is the number of cholesterols in the stationary state.

Integrating equation (6) we get:

$$N_{CHOL,c}(t) = (N_{CHOL,c}(0) - N^{s}_{CHOL,c})e^{-t(k_{ch}+k_{hc})} + N^{s}_{CHOL,c} \tag{8}$$

Alternatively, we can write the equations as:

$$N_{CHOL,c}(t) = N_{CHOL,c}(0) + (N^{s}_{CHOL,c} - N_{CHOL,c}(0))(1 - e^{-t(k_{ch}+k_{hc})}) \tag{9}$$

or in terms of the cholesterol fraction in the cold region, $x_{CHOL,c} = N_{CHOL,c}/N_{CHOL,T}$

Similarly:

$$x_{CHOL,c}(t) = x_{CHOL,c}(0) + (x^{s}_{CHOL,c} - x_{CHOL,c}(0))(1 - e^{-t(k_{ch}+k_{hc})}) \tag{10}$$

and $x_{CHOL,h}(t) = 1 - x_{CHOL,c}(t)$. Using $\tau = (k_{ch} + k_{hc})^{-1}$ we get equation (4) in the

main text.

Error analysis

All reported uncertainties correspond to statistical errors obtained from independent simulation repeats. For any primary observable X (e.g. bilayer thickness, heat flux, temperature gradient, thermal conductivity, thermal conductance, relaxation times τ, and the cholesterol fraction x_{chol}) measured over N independent runs, the uncertainty is defined as the sample standard deviation

$$\sigma_X = \sqrt{\frac{1}{N-1}\sum_{i=1}^{N}(X_i - \langle X\rangle)^2}, \qquad \langle X\rangle = \frac{1}{N}\sum_{i=1}^{N} X_i. \tag{11}$$

For derived quantities calculated from multiple independent observables, uncertainties were propagated assuming uncorrelated errors. The relative uncertainty for the thermal conductivity was computed as

$$\frac{\sigma_\lambda}{\lambda} = \sqrt{\left(\frac{\sigma_{J_q}}{J_q}\right)^2 + \left(\frac{\sigma_{\nabla T}}{\nabla T}\right)^2}. \tag{12}$$

The associated uncertainty for thermal conductance was given by

$$\frac{\sigma_G}{G} = \sqrt{\left(\frac{\sigma_\lambda}{\lambda}\right)^2 + \left(\frac{\sigma_\delta}{\delta}\right)^2}. \tag{13}$$

The uncertainties used in the error propagation of the Soret coefficient correspond to the standard deviations of the difference between cholesterol mole fractions in the hot and cold regions at steady-state, $\sigma_{\Delta x'_{CHOL}}$, and the temperature difference, $\sigma_{\Delta T}$, obtained from independent simulation repeats. The total uncertainty is given by (assuming $Cov(\Delta x, \Delta T) = 0$,

$$\frac{\sigma_{S_T}}{|S_T|} = \sqrt{\left(\frac{\sigma_{\Delta X}}{\Delta x}\right)^2 + \left(\frac{\sigma_{\Delta T}}{\Delta T}\right)^2} \tag{14}$$

All errors reported in the main text and Supporting Information represent one standard deviation ($\pm 1\sigma$) unless stated otherwise.

Table S6: RDF peak positions (r, nm) and heights ($g(r)$) for the first three maxima. For each temperature, the first row reports r and the second row reports $g(r)$; values are listed as (1st, 2nd, 3rd peak).

(a)

T (K)	Quantity	Pure (0%)	10% Chol	20% Chol
280	r	0.494, 0.896, 1.356	0.498, 0.984, 1.372	0.494, 0.980, 1.352
	$g(r)$	2.269, 1.348, 1.436	2.227, 1.308, 1.327	2.232, 1.314, 1.273
290	r	0.498, 0.924, 1.354	0.498, 0.960, 1.376	0.494, 0.944, 1.364
	$g(r)$	2.212, 1.309, 1.379	2.187, 1.322, 1.301	2.165, 1.294, 1.241
300	r	0.498, 0.958, 1.444	0.496, 0.960, 1.400	0.504, 0.976, 1.410
	$g(r)$	1.709, 1.173, 1.081	1.773, 1.188, 1.086	1.893, 1.231, 1.111
310	r	0.496, 0.972, 1.422	0.494, 0.970, 1.436	0.498, 0.976, 1.434
	$g(r)$	1.651, 1.153, 1.065	1.699, 1.163, 1.070	1.790, 1.190, 1.083
320	r	0.506, 0.970, 1.448	0.500, 0.978, 1.442	0.500, 0.976, 1.444
	$g(r)$	1.590, 1.135, 1.052	1.645, 1.155, 1.066	1.705, 1.162, 1.070
330	r	0.500, 0.974, 1.446	0.502, 0.974, 1.456	0.500, 0.980, 1.442
	$g(r)$	1.564, 1.117, 1.048	1.599, 1.129, 1.057	1.632, 1.144, 1.067
340	r	0.494, 0.970, 1.416	0.492, 0.982, 1.466	0.494, 0.976, 1.408
	$g(r)$	1.510, 1.105, 1.051	1.547, 1.116, 1.048	1.598, 1.124, 1.052

(b)

T (K)	Quantity	30% Chol	40% Chol	50% Chol
280	r	0.500, 0.970, 1.364	0.492, 0.974, 1.386	0.492, 0.964, 1.412
	$g(r)$	2.231, 1.289, 1.224	2.201, 1.286, 1.166	2.209, 1.253, 1.104
290	r	0.498, 0.968, 1.378	0.496, 0.968, 1.394	0.490, 0.988, 1.424
	$g(r)$	2.181, 1.294, 1.206	2.105, 1.272, 1.153	2.108, 1.236, 1.103
300	r	0.500, 0.974, 1.410	0.496, 0.942, 1.408	0.498, 0.980, 1.444
	$g(r)$	1.981, 1.252, 1.126	2.003, 1.244, 1.111	2.034, 1.225, 1.097
310	r	0.500, 0.978, 1.416	0.498, 0.984, 1.414	0.490, 0.976, 1.428
	$g(r)$	1.875, 1.226, 1.099	1.953, 1.224, 1.103	1.967, 1.224, 1.098
320	r	0.502, 0.986, 1.420	0.502, 0.992, 1.476	0.494, 0.986, 1.444
	$g(r)$	1.790, 1.197, 1.079	1.876, 1.208, 1.090	1.901, 1.227, 1.089
330	r	0.502, 0.990, 1.454	0.500, 1.002, 1.448	0.492, 0.964, 1.474
	$g(r)$	1.719, 1.166, 1.067	1.766, 1.187, 1.079	1.848, 1.198, 1.090
340	r	0.498, 0.970, 1.454	0.492, 0.980, 1.508	0.498, 0.970, 1.470
	$g(r)$	1.645, 1.147, 1.057	1.745, 1.168, 1.065	1.766, 1.174, 1.071

References

(S1) Bussi, G.; Donadio, D.; Parrinello, M. Canonical sampling through velocity rescaling. **2007**, *126*, 014101.

(S2) Bernetti, M.; Bussi, G. Pressure control using stochastic cell rescaling. *The Journal of Chemical Physics* **2020**, *153*, 114107.

(S3) Parrinello, M.; Rahman, A. Polymorphic transitions in single crystals: A new molecular dynamics method. *Journal of Applied Physics* **1981**, *52*, 7182–7190.

(S4) Abraham, M.; Alekseenko, A.; Andrews, B.; Basov, V.; Bauer, P.; Bird, H.; Briand, E.; Brown, A.; Doijade, M.; Fiorin, G.; Fleischmann, S.; Gorelov, S.; Gouaillardet, G.; Gray, A.; Irrgang, M. E.; Jalalypour, F.; Johansson, P.; Kutzner, C.; Łazarski, G.; Lemkul, J. A.; Lundborg, M.; Merz, P.; Miletić, V.; Morozov, D.; Müllender, L.; Nabet, J.; Páll, S.; Pasquadibisceglie, A.; Pellegrino, M.; Piasentin, N.; Rapetti, D.; Sadiq, M. U.; Santuz, H.; Schulz, R.; Shirts, M.; Shugaeva, T.; Shvetsov, A.; Turner, P.; Villa, A.; Wingbermühle, S.; Hess, B.; Lindahl, E. GROMACS 2025.3 Manual. **2025**, Publisher: Zenodo Version Number: 2025.3.

(S5) De Jong, D. H.; Singh, G.; Bennett, W. F. D.; Arnarez, C.; Wassenaar, T. A.; Schäfer, L. V.; Periole, X.; Tieleman, D. P.; Marrink, S. J. Improved Parameters for the Martini Coarse-Grained Protein Force Field. *J. Chem. Theory Comput.* **2013**, *9*, 687–697.

(S6) Tironi, I. G.; Sperb, R.; Smith, P. E.; Van Gunsteren, W. F. A generalized reaction field method for molecular dynamics simulations. *J. Chem. Phys.* **1995**, *102*, 5451–5459.

(S7) Humphrey, W.; Dalke, A.; Schulten, K. VMD: Visual molecular dynamics. *J. Mol. Graph.* **1996**, *14*, 33–38.

(S8) Brasnett, C.; Marrink, S. J. MartiniGlass: a Tool for Enabling Visualization of Coarse-Grained Martini Topologies. *J. Chem. Inf. Model.* **2025**, *65*, 3137–3141.

(S9) Smith, P.; Lorenz, C. D. LiPyphilic: A Python Toolkit for the Analysis of Lipid Membrane Simulations. *J. Chem. Theory Comput.* **2021**, *17*, 5907–5919.

(S10) Ramasubramani, V.; Dice, B. D.; Harper, E. S.; Spellings, M. P.; Anderson, J. A.; Glotzer, S. C. freud: A software suite for high throughput analysis of particle simulation data. *Comput. Phys. Commun.* **2020**, *254*, 107275.

(S11) Zhang, Y.; Lervik, A.; Seddon, J.; Bresme, F. A coarse-grained molecular dynamics investigation of the phase behavior of DPPC/cholesterol mixtures. *Chem. Phys. Lipids* **2015**, *185*, 88–98.

(S12) Carter, J. W.; Gonzalez, M. A.; Brooks, N. J.; Seddon, J. M.; Bresme, F. Flip-flop asymmetry of cholesterol in model membranes induced by thermal gradients. *Soft Matter* **2020**, *16*, 5925–5932.

(S13) Gittus, O. R.; Albella, P.; Bresme, F. Polarization of acetonitrile under thermal fields via non-equilibrium molecular dynamics simulations. *J. Chem. Phys.* **2020**, *153*, 204503.

(S14) Kučerka, N.; Nieh, M.-P.; Katsaras, J. Fluid phase lipid areas and bilayer thicknesses of commonly used phosphatidylcholines as a function of temperature. *Biochim. Biophys. Acta, Biomembr.* **2011**, *1808*, 2761–2771.

(S15) Youssefian, S.; Rahbar, N.; Lambert, C. R.; Van Dessel, S. Variation of thermal conductivity of DPPC lipid bilayer membranes around the phase transition temperature. *J. R. Soc. Interface.* **2017**, *14*, 20170127.

(S16) Luo, S.-N.; Strachan, A.; Swift, D. C. Nonequilibrium melting and crystallization of

a model Lennard-Jones system. *The Journal of Chemical Physics* **2004**, *120*, 11640–11649.

(S17) Pedersen, K. B.; Ingólfsson, H. I.; Ramirez-Echemendia, D. P.; Borges-Araújo, L.; Andreasen, M. D.; Empereur-Mot, C.; Melcr, J.; Ozturk, T. N.; Bennett, W. F. D.; Kjølbye, L. R.; Brasnett, C.; Corradi, V.; Khan, H. M.; Cino, E. A.; Crowley, J.; Kim, H.; Fábián, B.; Borges-Araújo, A. C.; Pavan, G. M.; Launay, G.; Lolicato, F.; Wassenaar, T. A.; Melo, M. N.; Thallmair, S.; Carpenter, T. S.; Monticelli, L.; Tieleman, D. P.; Schiøtt, B.; Souza, P. C. T.; Marrink, S. J. The Martini 3 Lipidome: Expanded and Refined Parameters Improve Lipid Phase Behavior. *ACS Cent. Sci.* **2025**, *11*, 1598–1610.

(S18) Zhao, H.; Bresme, F. Melting Point and Crystal Growth Kinetics of Metals and Metal Oxides Using Reactive Force Fields: The Case of Aluminum and Alumina. *Journal of Chemical Theory and Computation* **2024**, acs.jctc.4c00628.

(S19) Wang, Y.; Gkeka, P.; Fuchs, J. E.; Liedl, K. R.; Cournia, Z. DPPC-cholesterol phase diagram using coarse-grained Molecular Dynamics simulations. *Biochim. Biophys. Acta, Biomembr.* **2016**, *1858*, 2846–2857.

(S20) Vist, M. R.; Davis, J. H. Phase equilibria of cholesterol/dipalmitoylphosphatidylcholine mixtures: deuterium nuclear magnetic resonance and differential scanning calorimetry. *Biochemistry* **1990**, *29*, 451–464.

(S21) Koynova, R.; Caffrey, M. Phases and phase transitions of the phosphatidylcholines. *Biochim. Biophys. Acta, Rev. Biomembr.* **1998**, *1376*, 91–145.

(S22) Caffrey, M.; Hogan, J. LIPIDAT: A database of lipid phase transition temperatures and enthalpy changes. DMPC data subset analysis. *Chem. Phys. Lipids* **1992**, *61*, 1–109.

(S23) Winter, R.; Thiyagarajan, P. In *Trends in Colloid and Interface Science IV*; Zulauf, M., Lindner, P., Terech, P., Eds.; Steinkopff, 1990; Vol. 81; pp 216–221, Series Title: Progress in Colloid & Polymer Science.

(S24) Avanti Polar Lipids, Inc. Phase Transition Temperatures for Glycerophospholipids. `https://avantiresearch.com/tech-support/physical-properties/phase-transition-temps`, n.d.; Accessed: 2025-10-16. Compiled from Silvius, J. R. *Thermotropic Phase Transitions of Pure Lipids in Model Membranes and Their Modifications by Membrane Proteins*; Wiley, 1982, and the NIST LIPIDAT Database (SRD 34).

(S25) Stockton, G. W.; C.P. Smith, I. A deuterium nuclear magnetic resonance study of the condensing effect of cholesterol on egg phosphatidylcholine bilayer membranes. I. Perdeuterated fatty acid probes. *Chem. Phys. Lipids* **1976**, *17*, 251–263.

(S26) Rafieiolhosseini, N.; Ejtehadi, M. R. Thermal conductivity of the cell membrane in the presence of cholesterol and amyloid precursor protein. *Phys. Rev. E* **2020**, *102*, 042401.
